# Season‐specific impacts of climate change on canopy‐forming seaweed communities

**DOI:** 10.1002/ece3.10947

**Published:** 2024-02-13

**Authors:** Anthony T. Truong, Matthew S. Edwards, Jeremy D. Long

**Affiliations:** ^1^ Department of Biology San Diego State University San Diego California USA

**Keywords:** canopy‐forming species, climate change, community interactions, indirect effects, rocky intertidal, seasonality, understory assemblages

## Abstract

Understory assemblages associated with canopy‐forming species such as trees, kelps, and rockweeds should respond strongly to climate stressors due to strong canopy‐understory interactions. Climate change can directly and indirectly modify these assemblages, particularly during more stressful seasons and climate scenarios. However, fully understanding the seasonal impacts of different climate conditions on canopy‐reliant assemblages is difficult due to a continued emphasis on studying single‐species responses to a single future climate scenario during a single season. To examine these emergent effects, we used mesocosm experiments to expose seaweed assemblages associated with the canopy‐forming golden rockweed, *Silvetia compressa*, to elevated temperature and pCO_2_ conditions reflecting two projected greenhouse emission scenarios (RCP 2.6 [low] & RCP 4.5 [moderate]). Assemblages were grown in the presence and absence of *Silvetia*, and in two seasons. Relative to ambient conditions, predicted climate scenarios generally suppressed *Silvetia* biomass and photosynthetic efficiency. However, these effects varied seasonally—both future scenarios reduced *Silvetia* biomass in summer, but only the moderate scenario did so in winter. These reductions shifted the assemblage, with more extreme shifts occurring in summer. Contrarily, future scenarios did not shift assemblages within *Silvetia* Absent treatments, suggesting that climate primarily affected assemblages indirectly through changes in *Silvetia*. Mesocosm experiments were coupled with a field *Silvetia* removal experiment to simulate the effects of climate‐mediated *Silvetia* loss on natural assemblages. Consistent with the mesocosm experiment, *Silvetia* loss resulted in season‐specific assemblage shifts, with weaker effects observed in winter. Together, our study supports the hypotheses that climate‐mediated changes to canopy‐forming species can indirectly affect the associated assemblage, and that these effects vary seasonally. Such seasonality is important to consider as it may provide periods of recovery when conditions are less stressful, especially if we can reduce the severity of future climate scenarios.

## Graphical abstract

Climate change research has historically focused on single species responses to future climate conditions, leaving other factors that can alter these outcomes, such as climate scenario intensity, species interactions, and seasonality, understudied. We examined the effect of these additional factors on an intertidal rockweed community's response to climate change and found that different future climate scenarios interacted with the season, resulting in different rockweed communities.

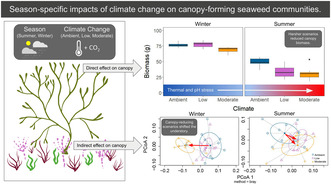

## 1 INTRODUCTION

Environmental stressors associated with climate change can influence the performance and survival of populations (Cavalcanti et al., 2018; Doney et al., 2020; Dudgeon, 2019; Heijmans et al., 2022; Kim et al., 2016; Spooner et al., 2018; Wernberg et al., 2011). When impacted populations are foundational, as with trees, rockweeds, kelps, corals, and seagrasses (Hoegh‐Guldberg, 1999; Hultine et al., 2020; Metzger et al., 2019; Sunny, 2017), these changes can alter community structure, ecosystem productivity, nutrient cycling, and energy flow (Boukal et al., 2019; Ehrenfeld, 2003; Lister & Garcia, 2018; Spector & Edwards, 2020; Sullaway & Edwards, 2020). The extent of these impacts, however, will depend on the severity of environmental change (i.e., which of the projected climate change scenarios actually occurs; Ángeles‐González et al., 2021; Reum et al., 2020), seasonal timing (i.e., how seasonal factors such as lighting and temperature interact with a changing climate; Ernakovich et al., 2014), and the characteristics of the species being considered (i.e., how species interactions shift under these climate change scenarios; Brown et al., 2014; Edwards, 2022; Kim et al., 2016). Unfortunately, the consequences of climate change on foundation species and their associated communities remain largely uncertain because most studies focus on the impacts of a single future climate scenario in a single season and a single population (Bass et al., 2021).

Simulating different future climate scenarios will better model climate change impacts by incorporating different levels of severity. The Intergovernmental Panel on Climate Change (IPCC) provided several Representative Concentration Pathways (RCP) that predict changes in temperature and ocean pH by the year 2100 relative to present‐day levels. For example, RCP 2.6 (+1°C/−0.1 pH units from ambient conditions) represents a low‐impact scenario where emissions are stabilized by the 2020s while RCP 4.5 (+2°C/−0.2 pH units) represents a moderate scenario where emissions are stabilized by the 2040s (IPCC, 2022). Observing the effects of climate change under multiple scenarios can reveal potential thresholds and offer greater predictability for management and conservation efforts (Thurman et al., 2020). Given (1) the uncertainty in the severity of future climate change, and (2) that small differences in temperature and/or pH can be biologically and ecologically meaningful (Araújo et al., 2018; Harrington et al., 2020; Wang et al., 2015), multiple scenarios need to be considered.

The severity of future climate change impacts on natural ecosystems may vary among seasons, but this variation also remains understudied (Russell et al., 2012). As future climate change continues to shift mean temperature and pH, anticipating how these shifts will vary between seasons is critical for timing‐dependent endeavors such as restorative transplanting (Li et al., 2014; Pearce‐Higgins et al., 2015; Richardson‐Calfee et al., 2004). Well‐known climate change alterations to seasonal events such as droughts, marine heatwaves, coastal upwelling, growing periods, and weather whiplash (i.e., rapid fluctuations between extreme conditions; Lee, 2022) have already disrupted phenological cycles and restructured communities across a wide range of ecosystems (Beas‐Luna et al., 2020; Bell et al., 2021; Donham et al., 2022; Ernakovich et al., 2014; Ooi et al., 2014). However, much of our understanding of species' responses to climate change derives from studies conducted under static laboratory conditions lacking seasonality (Kroeker et al., 2020). Including seasonal factors in climate change studies will provide clearer insights into performance impacts and mitigating these impacts.

Similar to studies performed under static conditions, population‐level studies have provided important insights (e.g., taxa‐specific effects of elevated pCO_2_; Cattano et al., 2018, Fernández et al., 2015, Kim et al., 2020, Lefevre, 2016). However, they may not accurately predict climate change impacts on whole communities because they do not allow for species interactions (Ockendon et al., 2014). Despite the staggering increase in climate change‐related research during the past two decades, the ratio of single‐species studies to community‐level studies remained nearly the same (i.e., single‐species studies continue to comprise ~60% of studies in this field, Bass et al., 2021). Additionally, when papers published between 2010 and 2019 were subdivided into those focusing on single species versus species assemblages, single species studies were three times more common (Bass et al., 2021; Wernberg et al., 2012). Reducing this gap and successfully predicting the impacts of climate change on natural populations will require increased efforts toward studying these impacts on natural assemblages.

Because canopy‐forming species interact strongly with their understory assemblages, climate change impacts may be particularly strong in communities containing such interactions (Edwards & Connell, 2012). Canopy‐forming species form densely branched overhead structures that modify the local environment (Edwards, 1998; Gonzales et al., 2017; Hondolero & Edwards, 2017; Joly et al., 2017; Ørberg et al., 2018). These effects can provide a more favorable habitat for understory species recruiting beneath or to the canopy (Clark et al., 2004; Flukes et al., 2014; Kitao et al., 2018; Roberts & Bracken, 2021). In turn, understory species can affect canopy‐forming species by augmenting the recruitment and survival of juvenile of canopy‐forming species (Barner et al., 2016; Beckley & Edwards, 2021). Under future climate conditions, these reciprocal interactions will likely alter the response of individual species to climate‐related stressors. For example, the canopy of giant kelp, *Macrocystis pyrifera*, may reduce the effects of climate change on understory species by absorbing excess CO_2_ (Hirsh et al., 2020). Consequently, the performance of understory species can be directly and/or indirectly affected by climate‐mediated changes (Edwards & Connell, 2012; Kim et al., 2020; Koch et al., 2013; Ragazzola et al., 2012). Given the complexity of these interactions, community‐level approaches should therefore be especially pertinent for these canopy‐dependent assemblages.

For this study, we identified a community that might be sensitive to the combined effects of climate change, seasonality, and species interactions. This community consisted of the canopy‐forming, intertidal, temperate rockweed, *Silvetia compressa* (henceforth *Silvetia*), and its understory assemblage. Rockweed canopies transform inhospitable intertidal areas such as exposed boulders into refuges by trapping moisture and stabilizing substrate and water temperature (Bertness et al., 1999). The assemblage associated with these refuges includes fleshy, turfing, and calcifying seaweeds, mobile and sessile intertidal invertebrates, and juvenile subtidal species such as fishes and lobsters (Sapper & Murray, 2003; Vercaemer et al., 2018). These understory species enhance primary productivity (Tait & Schiel, 2018), provide settlement cues and substrate for commercially important invertebrate larvae (Morse & Morse, 1984), and feed higher trophic levels (Ellis et al., 2007). Such interactions and corresponding services could be heavily altered should *Silvetia* populations decline. Recently, *Silvetia* declines have co‐occurred with ocean warming associated with the 2015–16 El Niño (Graham et al., 2018; MARINe, 2023). Future climate conditions resulting in similar levels of warming but across a prolonged period would likely exacerbate the decline of *Silvetia* communities.

Intertidal communities like this one might be particularly prone to season‐specific impacts of climate change for several reasons. First, future summers could become more stressful than other seasons in these habitats because intertidal organisms often live near their thermal maxima (Madeira et al., 2012). Second, tidal ranges are often season‐specific such that intertidal organisms will encounter more extreme conditions during these seasons (Erftemeijer & Herman, 1994; Flick, 2016). Third, seasonality may interact with climate in intertidal systems because the reproduction, dispersal, and recruitment of marine species are often seasonal (Ådahl et al., 2006; Edwards, 2022).

To understand the impacts of multiple climate change scenarios on *Silvetia* communities, we used mesocosms to expose *Silvetia* and its understory assemblages to three levels of ocean change conditions (Ambient, RCP 2.6, and RCP 4.5). These experiments also manipulated *Silvetia* presence to distinguish between the direct and indirect effects of climate change on the dominant understory species. We repeated this experiment in the summer and winter to assess seasonal variation in these effects. Because future climate scenarios were expected to suppress *Silvetia* growth, we also conducted field manipulations of *Silvetia* to understand the consequences of canopy loss on natural understory assemblages at two levels of understory biomass.

## 2 MATERIALS AND METHODS

### 2.1 Study site

**FIGURE 1:**
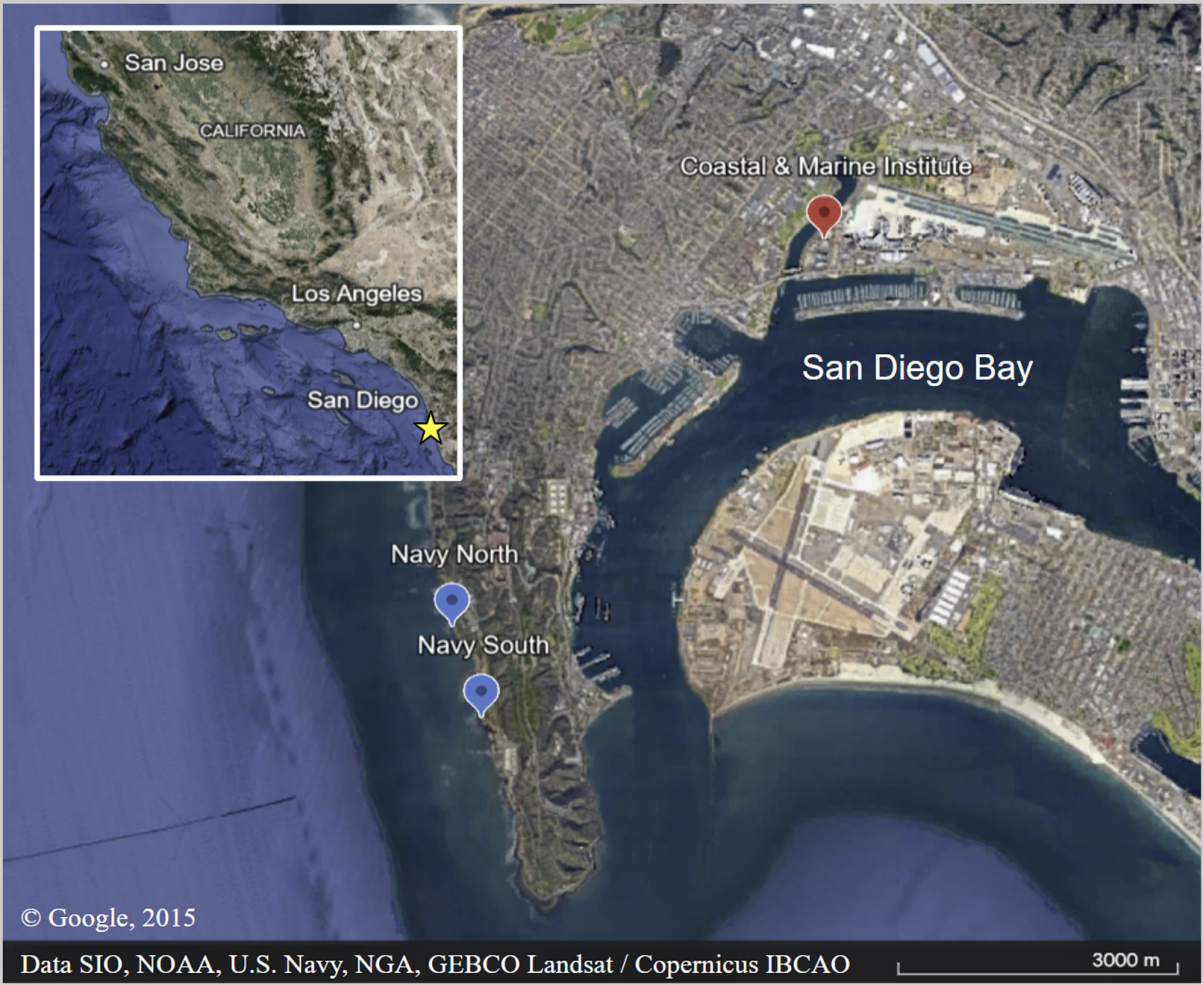
Map of Point Loma, San Diego (Google Maps, 2023). Algae and grazers were collected from Navy North and Navy South. Mesocosm experiments were conducted at the Coastal and Marine Institute. Field experiment plots were established at Navy South.

We selected sites adjacent to two southern California (Point Loma, San Diego, Figure 1) long‐term monitoring sites; Navy South (32.68306°N, −117.24963°W; hereafter NASO) and Navy North (32.692312°N, −117.25297°W; hereafter NANO). These Multi‐Agency Rocky Intertidal Network sites (MARINe) contain dense patches of *Silvetia*, perhaps because of the rarity of some stressors such as trampling (Denis, 2003; Tydlaska & Edwards, 2022) and runoff (Whitaker et al., 2010). We surveyed the *Silvetia* assemblages, collected the algae and grazers used in the mesocosm experiment, and conducted the field experiment at NASO. We added NANO as a secondary collection site to minimize the impact on algae and grazer populations at either site. Collecting procedures were identical between both sites. *Silvetia* at both sites grows on emergent substrata at intertidal elevations between 0 and 1 m above Mean Lower Low Water (hereafter MLLW). Average water temperatures at these sites are ~18°C and maximum summer water temperatures reach ~24°C (SeaTemperatures, 2023).

### 2.2 Mesocosm experiment

**FIGURE 2:**
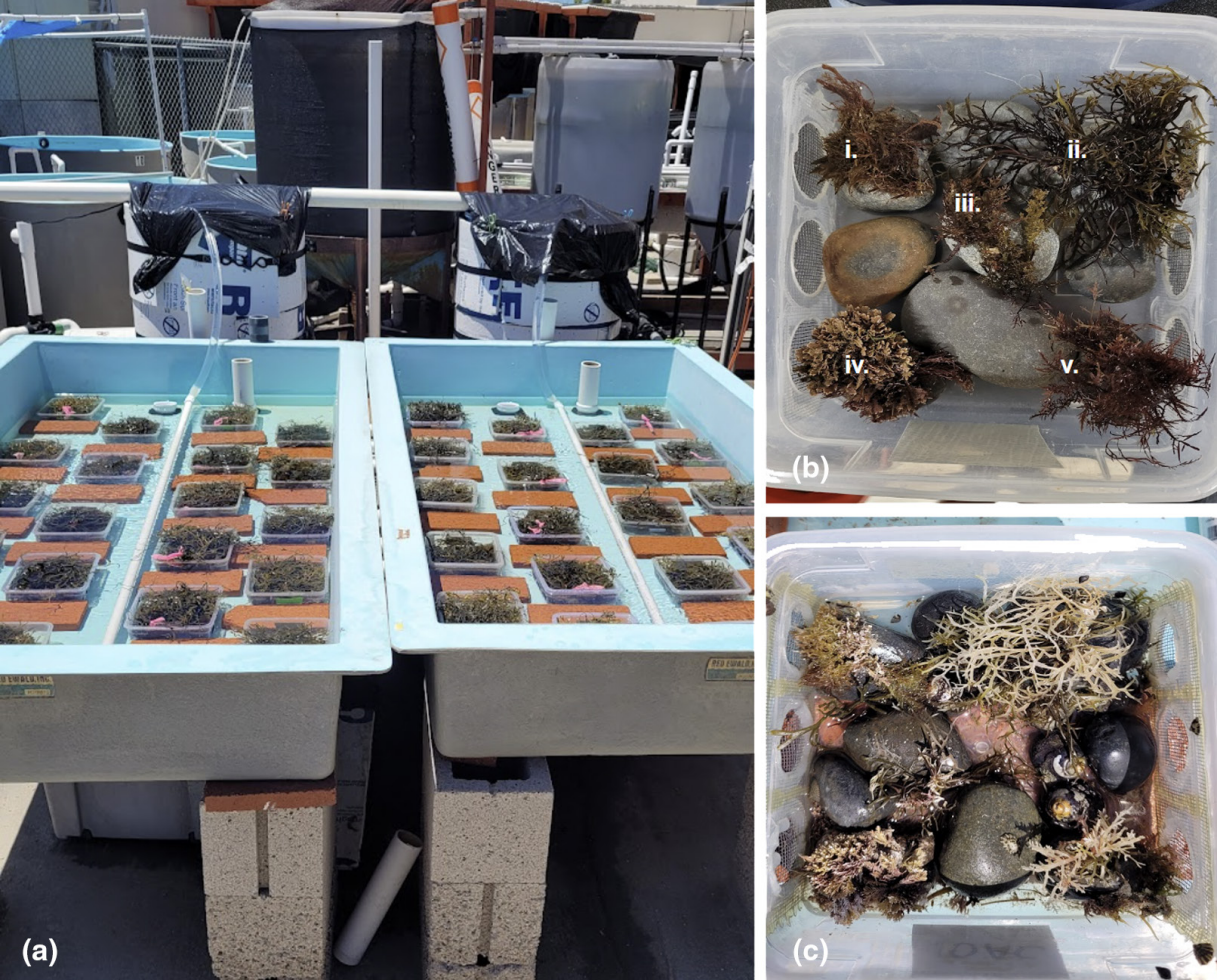
(a) Header tank and water table setup for the mesocosm experiment prior to removal of *Silvetia* canopies from the *Silvetia* Absent treatments and attachment of mesh lids. (b) An example of understory algae arrangement (i. *Centroceras*, ii. *Chondracanthus*, iii. *Laurencia*, iv. *Corallina*, v. *Gelidium*) within individual mesocosms. Note that due to availability, *Gelidium* was removed from the mesocosm experiment. (c) An example of understory algae bleaching over the course of the mesocosm experiment.

To examine the impacts of projected changes in ocean temperature and pH on *Silvetia* assemblages, we conducted a mesocosm experiment at San Diego State University's Coastal and Marine Institute (CMI, Figure 2a) that exposed the assemblages to three ocean climate conditions (Ambient, RCP 2.6, RCP 4.5). Ambient conditions represent current levels of temperature and pH. RCP 2.6 is a global emissions pathway representing low levels of climate change that will be experienced in the year 2100 (in line with the theoretical stabilization of global emissions by ~2020 leading to an average change of +1°C/−0.1 pH units on global oceans; IPCC, 2022). RCP 4.5 represents moderate levels of climate change (+2°C/−0.2 pH units). Importantly, our experiments used flow‐through seawater, which allowed for natural variation in ambient conditions. Thus, our future scenarios that manipulated pH and temperature relative to ambient conditions also experienced such variation.

Each mesocosm consisted of a clear polypropylene plastic box (15 × 15 × 7.6 cm; l × w × h) that had three 5‐cm diameter holes in each of two opposite sides. Window screen mesh covered these holes and the box tops to retain box contents and allow water exchange. We crossed climate scenario (Ambient, RCP 2.6, RCP 4.5) with *Silvetia* canopy (Present, Absent) treatments. Replicate mesocosms (*n* = 10) were randomly assigned to three outdoor water tables (1.8 × 0.9 × 0.3 m; l × w × h) that received flow‐through seawater from San Diego Bay. Each water table was then randomly assigned to one of the three climate scenarios. Because water temperatures in San Diego Bay are warmer than the average water temperatures at rocky shores where *Silvetia* occurs, we chilled the incoming seawater using a flow‐through seawater chiller (Aqualogic, Inc.) but still allowed the temperature to vary with natural ambient fluctuations (Figure S1). Seawater delivered to the future scenario mesocosms (i.e., RCP 2.6, RCP 4.5) was then altered in a header tank using aquarium heaters and CO_2_ injections to match the projected temperature and pH values of the respective scenarios before entering experimental mesocosms. Seawater was delivered to each header tank at 2270 L/h, which then flowed via gravity to the experimental mesocosms. To create realistic tidal conditions, ball valves connected to drains were opened and closed using a digital watering timer (DIG Model C002, DIG Corporation), which resulted in the mesocosms being submerged at tide heights 0.5 m above MLLW, and emerged at tide heights below this. This tide height is representative of intertidal elevations where *Silvetia* occurs in southern California (Littler, 1980).

We added realistic assemblages of understory algae to each mesocosm. To determine the species that comprised these representative assemblages, we surveyed natural understory algal communities in the field at NASO. We collected, identified, and weighed all the understory algae found within six 0.15 × 0.15 m quadrats that were placed beneath haphazardly selected *Silvetia* individuals. This identified five genera that made up 83% of the total understory algal biomass; namely *Chondracanthus*, *Centroceras*, *Corallina*, *Gelidium*, and *Laurencia* (Figure 2b). Because we were unable to find enough *Gelidium* during future collections for our experiment, we removed it from the study. The remaining four genera made up 76% of total understory biomass. To create realistic understory assemblages, we calculated the biomass density of each genera in the field (grams per m^2^) and scaled these calculations to match the surface area of the mesocosm floors. Using this approach, each mesocosm received 4 g of *Centroceras*, 10 g of *Chondracanthus*, 9 g of *Corallina*, and 2.5 g of *Laurencia*. Additionally, because invertebrate grazers can alter algal‐algal interactions such as by controlling epiphyte growth (Hoffmann et al., 2020; Rogers & Breen, 1983), they were included in all mesocosms. To add ecologically realistic densities of these grazers relative to *Silvetia* biomass, we scaled field densities (# of grazer individuals per gram of *Silvetia*) reported in a previous study (Jones, 2016) to our mesocosms. As a result, we added six *Tegula funebralis*, six *Lottia strigatella*, six *Lottia scabra*, 10 *Littorina scutulata*, and one *Cyanoplax hartwegii* to each mesocosm.

Grazers and understory algae were collected during the establishment of the field experiment (see below) and held at the CMI for a 10‐day acclimation period. Each of the four understory seaweeds was weighed to the predetermined biomass (±5%), attached to a rock with superglue, and placed into one of the four corners of the mesocosms. Additional rocks covered the bottom of the mesocosms to provide a refuge for grazers. We alternated the position of each algal type between replicates in a Latin Square design. For the *Silvetia* Present treatments, pre‐weighed *Silvetia* (72.0 ± 5% g) were laid across the assemblage inside mesocosm containers. Actual average *Silvetia* weight was 72.5 ± 1.3 g (mean ± SE). Once assembled, the mesocosms were placed into the water tables containing ambient seawater. Header tanks containing treated seawater would then alter the pH and temperature of the experimental tables to match treatment values over the course of approximately 1 day. During our 42‐day experiment (August 5th–September 17th, 2021), we measured the pH and temperature of the seawater as it flowed from each header tank into the experimental mesocosms every morning using a probe (Oakton 300 Series pH/DO meter), except on days 32, 38, and 39, which were not measured due to logistical constraints.

After 42 days, we ended this experiment as most of the understory algae in the *Silvetia* Absent treatments had bleached or disintegrated (Figure 2c). We categorized the algae tissue as being either bleached (dead) or unbleached (living) and measured the biomass of each group in each replicate after blotting them dry. After separating the bleached tissue, the remaining biomass of each understory genus was then calculated as the percentage of final unbleached tissue weight relative to its initial weight. To assess *Silvetia* health, we measured quantum yield (a ratio of variable fluorescence [Fv] to maximal fluorescence [Fm]), which estimates the light‐harvesting efficiency of photosystem II (PS II), using a pulse amplitude modulated (PAM) fluorometer (sensu Bews et al., 2021; Edwards & Kim, 2010). Because we observed within‐individual variation in tissue health, we measured the quantum yield of each individual at five randomly selected sections of each thallus and averaged these measurements for each *Silvetia* replicate.

To understand seasonal differences in how the *Silvetia* assemblage responded to climate change, we repeated this experiment in the winter (November 9–December 20, 2021). We followed the same protocols described above but made three changes: (1) We shortened the acclimation period from 10 to 5 days, (2) replaced all algae and grazers with newly collected individuals from a nearby site (NANO instead of NASO), and (3) the water tables were randomly reassigned different climate treatments. Pre‐weighed *Silvetia* for this experiment averaged 71.0 ± 1.6 g. Although we did not see as much understory degradation in the *Silvetia* Absent treatments during this experiment, we maintained the 42‐day experimental duration to facilitate comparisons between the two trials (hereafter summer and winter).

### 2.3 Field experiment

Experimental field plots were established at NASO to simulate the effect of climate change‐mediated loss of *Silvetia* on its understory assemblage. Because the effect of canopy loss on the assemblage could depend upon the successional stage of the assemblage, we also manipulated the assemblage biomass of the understory by clearing half of the plots at the start of the experiment. We crossed *Silvetia* Canopy (High, Partial, None) with the initial state of the Understory (Full, Cleared); *n* = 10. We established these plots in the summer (July 2021) because we hypothesized that the effects of *Silvetia* loss should be most pronounced during the less favorable summer conditions. Plots containing *Silvetia* (0.15 × 0.15 m) were marked at their corners with Z‐spar Splash Zone epoxy and were randomly assigned to the different treatments. Plots were positioned just below the existing *Silvetia* holdfasts to study the understory species beneath where the *Silvetia* canopy drapes over the substrate during low tide. Prior to manipulations, we recorded the percent cover of each genus within the plots using 25‐point intercepts within 0.15 × 0.15 m quadrats.

Plots assigned to the *Silvetia* Canopy None treatments simulated the effects of climate change‐mediated loss of *Silvetia* by trimming *Silvetia* to its holdfast using shears. This allowed the thallus to eventually regrow, while still subjecting the assemblage to any effects associated with an absent canopy for the duration of the experiment. In previous mesocosm experiments, future climate conditions caused *Silvetia* to discolor, shrivel, and lose biomass across its entire thalli (J.D. Long, 2015, unpublished data). To examine the consequences of partial *Silvetia* loss, we trimmed *Silvetia* in Partial Canopy treatments from multiple layers originating from a single holdfast to a single thallus layer. The remaining plots containing *Silvetia* were left unmanipulated and represented our High Canopy treatments. However, because (1) we observed large within‐treatment variation and (2) the *Silvetia* Canopy High and Partial treatments provided similar canopies, we pooled High and Partial *Silvetia* treatments into a single “*Silvetia* Present” treatment (*n* = 20) and compared this pooled treatment to the *Silvetia* Canopy None (henceforth “*Silvetia* Absent”) treatment; *n* = 10. Because we were unable to relocate some plots during subsequent surveys, our final samples varied from 14 to 16 and 6 to 8 for *Silvetia* Present and *Silvetia* Absent treatments, respectively. To manipulate the understory assemblages, the existing assemblages in half of the plots of each *Silvetia* treatment were removed using scrapers and chisels (Understory Cleared treatments) while the assemblages in the other half were left unmanipulated (Understory Full treatments). We measured the percent cover of the understory assemblages in October (hereafter fall) and December (hereafter winter) 2021.

### 2.4 Statistical analyses

All data were analyzed using R‐Studio and Primer + PERMANOVA 7. Prior to analyses, data were checked for normality and heteroscedasticity using Shapiro–Wilk's and Levene's tests, respectively.

For the mesocosm experiment, measurements of quantum yield required square‐root transformation to meet assumptions of normality. *Silvetia* biomass and measurements of quantum yield within the mesocosms were compared among the three climate treatments using separate one‐way ANOVAs (for each season). This was done as separate analyses rather than a two‐way ANOVA that included season as a factor because the experimental mesocosms were broken down, cleaned, randomized, and reassigned with new assemblages prior to the winter trial. Tukey's HSD post hoc tests between pairs of climate treatments were then used when the ANOVAs returned significant differences. To visualize shifts in the understory algal assemblages between the Climate and *Silvetia* canopy treatments within each trial, Principal Coordinates Analysis (PCoA) based on Bray–Curtis dissimilarity matrices was used to map similarities in the algae comprising each assemblage. Two‐way PERMANOVAs were then used to determine if the assemblage shifts differed between the Climate and *Silvetia* canopy treatments. Due to a high number of zeroes for certain taxa in the *Silvetia* Absent treatments, the data were square‐root transformed and the PERMANOVAs were run with a zero‐inflated Bray–Curtis similarity indices using a dummy variable of 1 (Clarke et al., 2006; Smith, 2017). A priori post‐hoc permutation tests were then used to examine pairwise differences in the assemblages between Climate and *Silvetia* canopy treatments. SIMPER analyses were used to identify the relative contribution of each understory taxon to assemblage dissimilarity between treatments. As discussed above, these analyses were run separately for the summer and winter trials.

For the field experiment, a three‐way PERMANOVA was used to assess differences in the understory communities (based on percent cover) between *Silvetia* canopy treatments, Understory treatments, and Seasons. Unlike the mesocosm experiments, the season was included as a factor because the field experiment was run continuously. Due to consolidating *Silvetia* High and Low plots into a single treatment, our experimental design was unbalanced, and this was compounded by the loss of plots due to storms. PERMANOVA is the most robust test under this design, but because it still loses reliability with increasing heterogeneity, we acknowledge the potential statistical error for this analysis (Anderson & Walsh, 2013). Following the PERMANOVA, a priori permutation post‐hoc tests were used to determine differences in understory assemblages between the *Silvetia* canopy treatments within each Understory treatment and season. SIMPER analyses were used to determine the percent contribution of each general to the observed differences. All analyses were evaluated at an *α*‐level of .05.

## 3 RESULTS

### 3.1 Mesocosm conditions

Seawater conditions within the climate treatments representing future climate scenarios approximated the desired target values for temperature and pH of +1°C and –0.1 pH units (RCP 2.6), and +2°C and –0.2 pH units (RCP 4.5) relative to Ambient conditions. In summer, average RCP 2.6 parameters measured +1.2°C and –0.16 pH units while average RCP 4.5 parameters measured +1.8°C and –0.25 pH units. In winter, average RCP 2.6 parameters measured +0.6°C and –0.14 pH units while average RCP 4.5 parameters measured +1.8°C and –0.22 pH units (Figure S1, Table S1). On average, all three treatments varied with natural ambient fluctuations and were warmer and more acidic during the summer trial than during the winter trial.

### 3.2 *Silvetia* biomass

**FIGURE 3:**
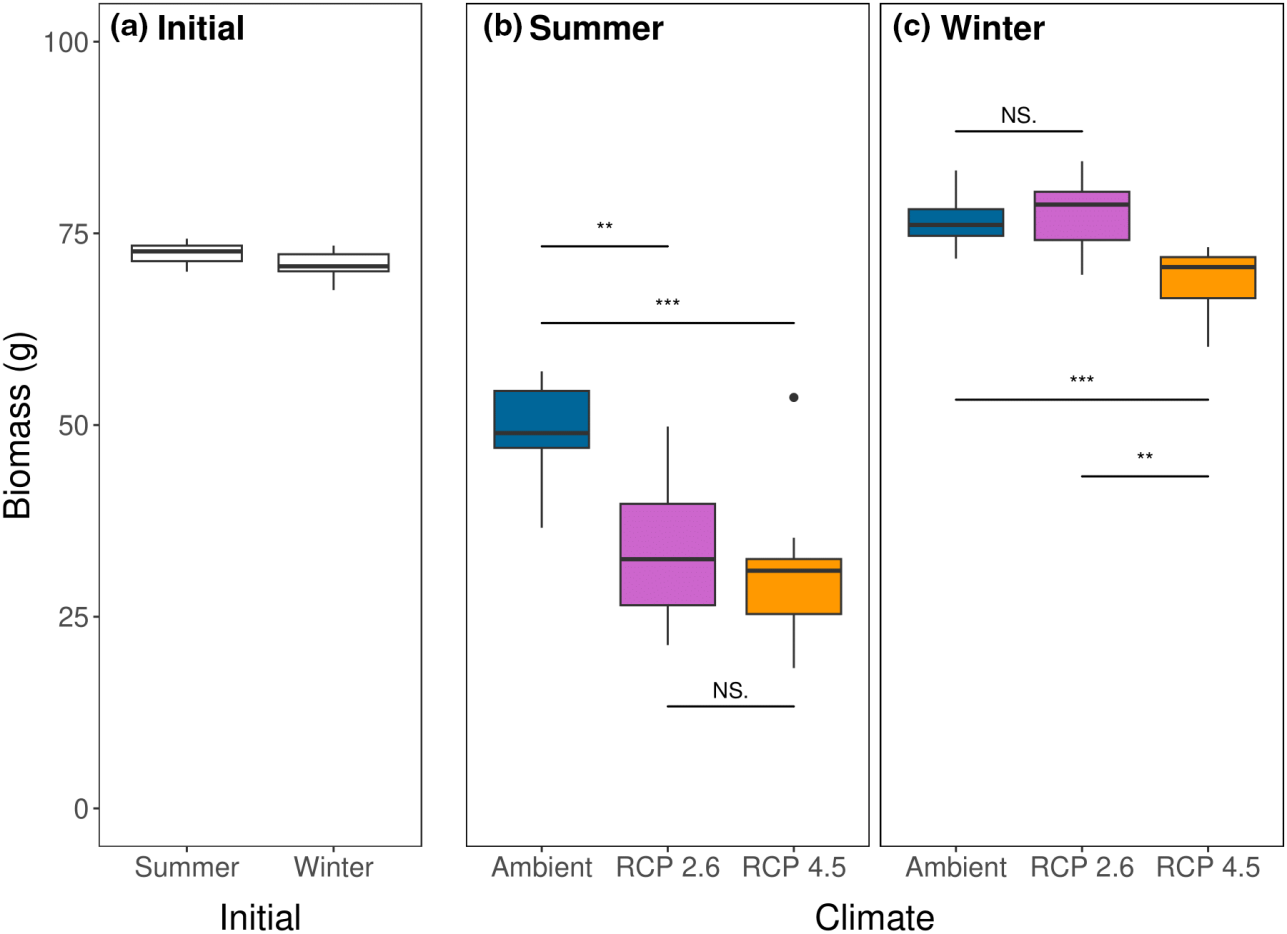
Box and whisker plots representing (a) initial *Silvetia* biomass and final *Silvetia* biomass between Climate treatments for the (b) summer trial and (c) winter trial of the mesocosm experiment. Horizontal lines and asterisks between Climate treatments denote levels of significant differences within each trial (NS, not significant).

The climate change treatments within the mesocosms affected final *Silvetia* biomass in both the summer (ANOVA: *F*_2,27_ = 11.4, *p* < .001) and winter (*F*_2,27_ = 13.4, *p* < .001, Figure 3, Table S2). Specifically, during the summer trial, *Silvetia* biomass declined in all three climate treatments relative to starting biomass (Tukey's: *p* < .001 for all), with more pronounced declines under both future climate scenarios relative to Ambient conditions (Tukey's: *p* = .003 & *p* < .001 for RCP 2.6 & RCP 4.5, respectively). However, *Silvetia* biomass loss did not differ between the two future climate scenarios (Tukey's: *p* = .710). In contrast, *Silvetia* biomass increased significantly under Ambient (*p* = .004) and RCP 2.6 (*p* < .001) conditions relative to its starting biomass during the winter trial but did not change under RCP 4.5 conditions (*p* = .467). Consequently, biomass under Ambient and RCP 2.6 conditions remained similar to one another (*p* = .828) in the Ambient and RCP 2.6 mesocosms but were both higher than in the RCP 4.5 mesocosms (*p* < .001 for both). Overall, final *Silvetia* biomass was higher in the winter trial (74.2 ± 5.6 g, mean ± SE) relative to the summer trial (37.8 ± 11.8 g, mean ± SE). When comparing the same climate treatments across seasons (e.g., Ambient in summer to Ambient in winter), the biomass of all three winter treatments was higher than those of the summer treatments (Figure 3).

### 3.3 *Silvetia* quantum yield

**FIGURE 4:**
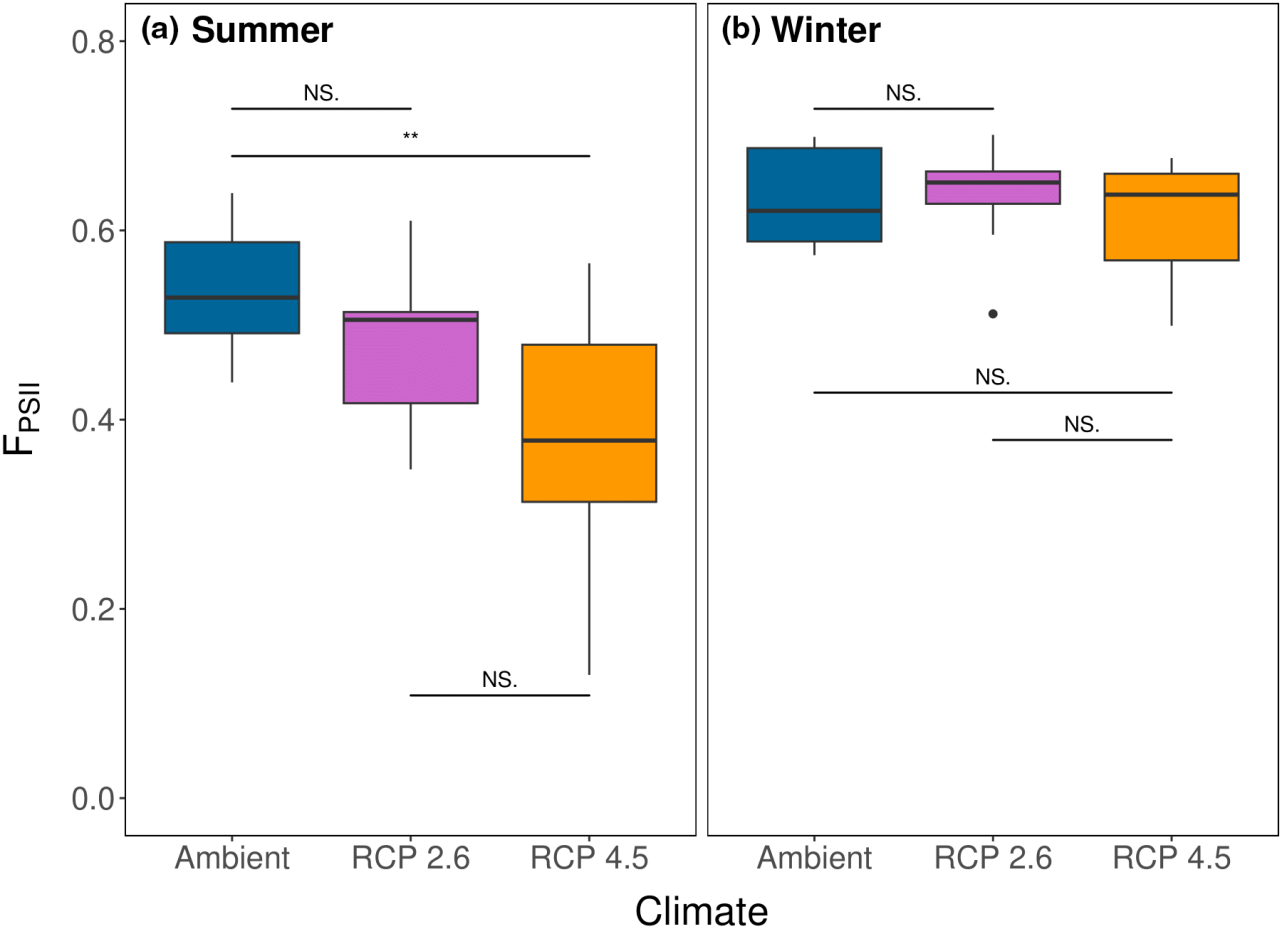
Box and whisker plots representing the final *Silvetia* quantum yield between Climate treatments for the (a) summer trial and (b) winter trial of the mesocosm experiment. Horizontal lines and asterisks between Climate treatments denote levels of significant differences within each trial (NS, not significant).

Similar to biomass, *Silvetia* quantum yield varied among the climate treatments in both summer (ANOVA: *F*_2,27_ = 6.5, *p* = .005) and winter (*F*_2,27_ = 0.5, *p* = .635, Figure 4, Table S3), but this appeared to differ between the two seasons. Specifically, quantum yield was generally higher in the winter (0.63 ± 0.05 Φ_PSII_, mean ± SE) than in the summer (0.46 ± 0.12 Φ_PSII_, mean ± SE). In summer, *Silvetia* quantum yield varied among climate change treatments and was significantly lower under RCP 4.5 conditions relative to Ambient conditions (Tukey's: *p* = .004), but otherwise, it did not differ between Ambient and RCP 2.6 conditions (*p* = .302) or between RCP 2.6 versus RCP 4.5 conditions (*p* = .115). In contrast, the quantum yield did not vary among the climate treatments in the winter trial (ANOVA: *F*_2,27_ = 0.5, *p* = .635), though the quantum yield of every winter climate treatment was higher than the summer counterpart (Figure 4).

### 3.4 Mesocosm assemblages

**FIGURE 5:**
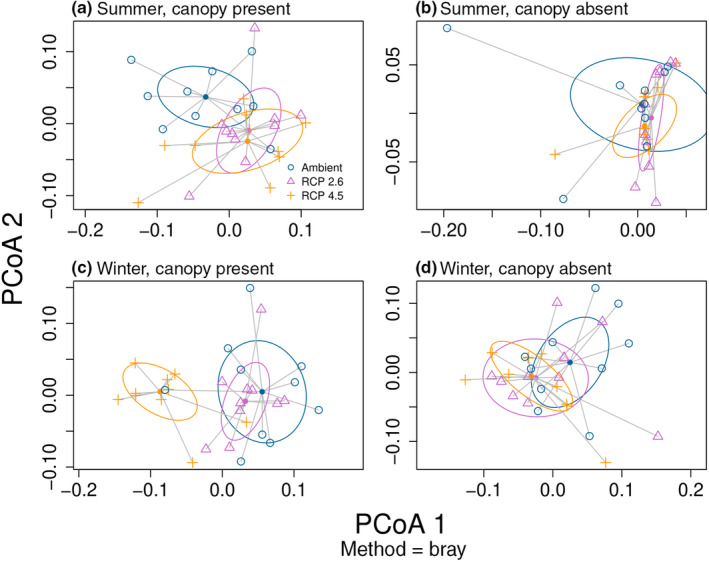
Principal Coordinates Analysis (PCoA) plots depicting the change in the understory assemblage across Climate and Canopy treatments for the mesocosm experiment. The assemblage composition of (a) *Silvetia* Canopy Present treatments during the summer, (b) *Silvetia* Canopy Absent treatments during the summer, (c) *Silvetia* Canopy Present treatments during the winter, and (d) *Silvetia* Canopy Absent treatments during the winter. Assemblage composition of each plot was generated using the biomass of each algal genera recovered at the end of each trial.

When climate change treatments reduced *Silvetia* biomass relative to Ambient in the *Silvetia* Present treatments, significant changes to the associated understory assemblage followed (PERMANOVA: pseudo‐*F*_2,54_ = 2.8 & 1.7, *p* = .001 & .156 for summer and winter [differences found via a priori testing], respectively; Figure 5, Table S4). Specifically, during the summer trial when the biomass of the *Silvetia* canopy was reduced under future climate scenarios, the understory communities shifted relative to Ambient (Pairwise tests: *p* = .012 & .013 for comparisons of RCP 2.6 & RCP 4.5 to Ambient, respectively, Figure 5a, Table S5). However, the understory assemblages were not different between RCP 2.6 and RCP 4.5 (*p* = .583). In contrast, no shifts in the understory assemblages occurred under either climate change scenario relative to Ambient in the absence of the *Silvetia* canopies (Pairwise tests: *p* = .165 & .420 for comparisons of RCP 2.6 & RCP 4.5 to Ambient, respectively, Table S5), and the understory assemblages were again not different between RCP 2.6 and RCP 4.5 (*p* = .460, Figure 5b). Similarly, understory shifts during the winter trial occurred in the presence of a *Silvetia* canopy, but only under RCP 4.5 conditions (i.e., when *Silvetia* biomass was lower than it was in Ambient & RCP 2.6 treatments, Pairwise tests: *p* = .001 for both, Figure 5c). In the absence of a canopy, like the summer trial, there were no differences between understory assemblages of either climate change scenario relative to Ambient (Pairwise tests: *p* = .831 & .065 for comparisons of RCP 2.6 & RCP 4.5 to Ambient, respectively, Table S5) or between RCP 2.6 and RCP 4.5 (*p* = .655).

When future climate scenarios shifted the understory assemblage beneath a *Silvetia* canopy compared to the Ambient treatment (i.e., RCP 2.6 & RCP 4.5 in summer, and RCP 4.5 in winter), we observed the same ranking in taxa with respect to their contribution to this dissimilarity. From most important to least important, this ranking was the same in these three comparisons—*Centroceras*, *Corallina*, *Chondracanthus*, *Laurencia* (Table 1, taxa rankings for *Silvetia* Absent treatments can be found in Table S6).

**TABLE 1:** Cumulative percent contribution, generated by SIMPER, of each algal genera to assemblage dissimilarity between Climate treatments within the *Silvetia* Canopy Present treatment of the mesocosm experiment.

Ambient versus RCP 2.6 (summer, canopy present)									Ambient versus RCP 2.6 (winter, canopy present)								
Average dissimilarity: 0.25									Average dissimilarity: 0.22								
Genus	Ambient	RCP 2.6	Av.Diss	SD	Ratio	Contrib. %	Cum. %	*p*	Genus	Ambient	RCP 2.6	Av.Diss	SD	Ratio	Contrib. %	Cum. %	*p*
	Av.Abund	Av.Abund								Av.Abund	Av.Abund						
*Centroceras*	44.31	33.39	0.10	0.08	1.21	39.8	39.8	.527	*Centroceras*	56.23	51.60	0.08	0.06	1.42	35.8	35.8	.725
*Corallina*	33.38	16.51	0.08	0.04	1.84	34.3	74.1	.003	*Chondracanthus*	40.64	37.45	0.06	0.04	1.57	27.6	63.4	.941
*Chondracanthus*	20.67	19.43	0.06	0.05	1.17	23.2	97.3	.892	*Laurencia*	43.28	36.45	0.04	0.04	1.14	18.9	82.3	.166
*Laurencia*	28.32	29.27	0.01	0.01	0.81	2.7	100.0	.742	*Corallina*	58.18	51.08	0.04	0.03	1.23	17.7	100.0	1.000

Ambient versus RCP 4.5 (summer, canopy present)									Ambient versus RCP 4.5 (winter, canopy present)								
Average dissimilarity: 0.26									Average dissimilarity: 0.33								
Genus	Ambient	RCP 4.5	Av.Diss	SD	Ratio	Contrib. %	Cum. %	*p*	Genus	Ambient	RCP 4.5	Av.Diss	SD	Ratio	Contrib. %	Cum. %	*p*
	Av.Abund	Av.Abund								Av.Abund	Av.Abund						
*Centroceras*	44.31	36.01	0.10	0.08	1.23	39.4	39.4	.323	*Centroceras*	56.23	29.33	0.10	0.07	1.36	30.6	30.6	.029
*Corallina*	33.38	16.51	0.08	0.04	1.80	30.4	69.8	.005	*Corallina*	58.18	31.46	0.09	0.05	1.93	28.3	58.9	.001
*Chondracanthus*	20.67	28.45	0.07	0.06	1.19	25.8	95.6	.501	*Chondracanthus*	40.64	20.12	0.09	0.07	1.24	26.8	85.7	.017
*Laurencia*	28.32	30.08	0.01	0.03	0.44	4.4	100.0	.668	*Laurencia*	43.28	28.22	0.05	0.05	0.99	14.3	100.0	.024

RCP 2.6 versus RCP 4.5 (summer, canopy present)									RCP 2.6 versus RCP 4.5 (winter, canopy present)								
Average dissimilarity: 0.21									Average dissimilarity: 0.28								
Genus	RCP 2.6	RCP 4.5	Av.Diss	SD	Ratio	Contrib. %	Cum. %	*p*	Genus	RCP 2.6	RCP 4.5	Av.Diss	SD	Ratio	Contrib. %	Cum. %	*p*
	Av.Abund	Av.Abund								Av.Abund	Av.Abund						
*Centroceras*	33.39	36.01	0.08	0.07	1.28	39.9	39.9	.837	*Centroceras*	51.60	29.33	0.09	0.07	1.32	32.3	32.3	.217
*Chondracanthus*	19.43	28.45	0.07	0.06	1.28	35.2	75.1	.131	*Chondracanthus*	37.45	20.12	0.08	0.04	1.93	29.0	61.3	.050
*Corallina*	16.51	16.51	0.04	0.04	1.00	17.6	92.7	1.000	*Corallina*	51.08	31.46	0.08	0.04	1.82	28.6	89.9	.005
*Laurencia*	29.27	30.08	0.02	0.03	0.57	7.3	100.0	.064	*Laurencia*	36.45	28.21	0.03	0.04	0.69	10.1	100.0	.878

*Note*: Left column: Summer trial. Right column: Winter trial. The average ratio of final recovered biomass to initial biomass of each algal genera was used for the analysis of dissimilarity.

In the summer and relative to Ambient climate treatments, 40% of the dissimilarity with RCP 2.6 treatments was driven by a decrease in *Centroceras* (44.3 ± 27.5%–33.4 ± 14.7%, mean ratio of recovered biomass to initial biomass ± SE). In addition, 34% of the dissimilarity was driven by a decrease in *Corallina* (from 33.4 ± 11.5% to 16.5 ± 8.9%), 23% by a decrease in *Chondracanthus* (20.7 ± 13.6%–19.4 ± 12.2%), and lastly, 3% by an increase in *Laurencia* (from 28.3 ± 0.7% to 29.3 ± 2.0%). For summer RCP 4.5, a decrease in *Centroceras* (44.3 ± 27.5%–36.0 ± 21.9%) and *Corallina* (33.4 ± 11.5%–16.5 ± 7.0%) drove 40% and 30% of the dissimilarity, respectively, while an increase in *Chondracanthus* (20.7 ± 13.6%–28.4 ± 18.8%) and *Laurencia* (28.3 ± 0.7%–30.1 ± 5.8%) drove 26% and 4% of the dissimilarity, respectively. Lastly, in winter and relative to Ambient, all genera in RCP 4.5 decreased with 31% of the dissimilarity driven by *Centroceras* (56.2 ± 27.8%–29.3 ± 9.6%), 28% by *Corallina* (58.2 ± 11.7%–31.5 ± 11.3%), 27% by *Chondracanthus* (40.6 ± 25.1%–20.1 ± 14.8%), and 14% by *Laurencia* (from 43.4 ± 16.9% to 28.2 ± 0.4%, Table S7).

Overall, the top two genera (*Centroceras* and *Corallina*) consistently declined under future climate scenarios relative to Ambient climates in these three treatments (RCP 2.6 & RCP 4.5 in summer, and RCP 4.5 in winter, Figure S2). The other two genera showed more variable responses to these treatments. For example, *Chondracanthus* decreased in two of these treatments (summer, RCP 2.6 and winter, RCP 4.5) but increased in another (summer, RCP 4.5). Similarly, *Laurencia* decreased in one of these treatments (winter, RCP 4.5) but increased in two other treatments (summer, RCP 2.6 and summer, RCP 4.5).

### 3.5 Field assemblage

**FIGURE 6:**
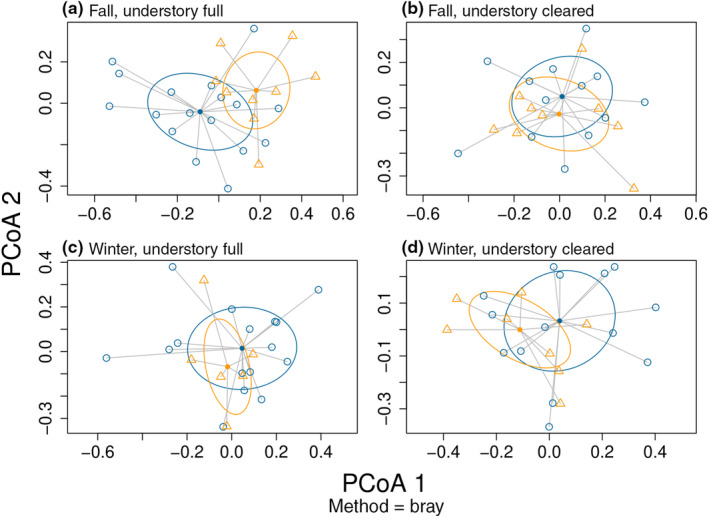
Principal Coordinates Analysis (PCoA) plots depicting shifts in the understory assemblage between *Silvetia* Canopy and Understory treatments for the field experiment based on surveys conducted in fall and winter. The assemblage composition for (a) fall, Understory Full treatments, (b) fall, Understory Cleared treatments, (c) winter, Understory Full treatments, and (d) winter, Understory Cleared treatments. Assemblage composition of each plot was generated using the percent cover of each algal genera.

The effect of *Silvetia* removal on the understory assemblages in our field plots depended upon season and the initial state of the understory assemblages (PERMANOVA: pseudo‐*F*_1,84_ = 3.6, *p* = .002, Figure 6, Table S10). Specifically, in fall, the *Silvetia* Canopy treatment influenced the assemblage in the Understory Full treatments (Pairwise tests: *p* = .009, Figure 6a, Table S11). Under this scenario, the greatest contributors to dissimilarity (listed in order of importance, Table 2) were *Centroceras* (~17%), *Corallina* (~16%), *Laurencia* (~13%), Bare Rock (~10%), *Gigartina* (~10%), and *Gelidium* (~9%). Importantly, the top two species that responded to *Silvetia* loss were *Centroceras* and *Corallina* were the same top two species that were impacted by the climate manipulations in our mesocosm experiment. *Silvetia* absence was associated with an increase in the average percent cover of *Centroceras* (from 7.8 ± 13.7% to 25.3 ± 18.2%) while these conditions led to a decrease in *Corallina* (from 25.2 ± 20.3% to 11.6 ± 13.2%). *Laurencia* also increased in the absence of a canopy (from 10.0 ± 15.7% to 17.3 ± 19.8%), as did *Gigartina* (from 7.1 ± 13.1% to 10.2 ± 16.3%) and Bare Rock (from 9.2 ± 9.5% to 14.7 ± 16.5%), while *Gelidium* declined (from 12.3 ± 20.4% to 0.0 ± 0.0%) under these conditions (Table S13, Figure S4).

**TABLE 2:** Cumulative percent contribution for each algal genera, generated by SIMPER, to significant assemblage dissimilarity in the field experiment between *Silvetia* Present and *Silvetia* Absent treatments.

Canopy present versus canopy absent (fall, understory full)									Canopy present versus canopy absent (winter, understory cleared)								
Average dissimilarity: 0.50									Average dissimilarity: 0.44								
Genus	Present	Absent	Av.Diss	SD	Ratio	Contrib. %	Cum. %	*p*	Genus	Present	Absent	Av.Diss	SD	Ratio	Contrib. %	Cum. %	*p*
	Av.Abund	Av.Abund								Av.Abund	Av.Abund						
*Centroceras*	0.08	0.25	0.11	0.08	1.37	16.8	16.8	.009	Bare Rock	0.16	0.43	0.14	0.08	1.68	23.8	23.8	.001
*Corallina*	0.26	0.12	0.11	0.09	1.24	15.7	32.5	.481	*Centroceras*	0.17	0.18	0.11	0.08	1.27	17.9	41.7	.731
*Laurencia*	0.10	0.17	0.09	0.09	1.02	13.3	45.8	.188	*Corallina*	0.15	0.17	0.08	0.05	1.55	13.4	55.1	.674
Bare Rock	0.09	0.15	0.07	0.07	1.05	10.1	55.9	.205	*Gigartina*	0.11	0.05	0.06	0.08	0.71	10.2	65.3	.682
*Gigartina*	0.07	0.10	0.06	0.08	0.83	9.6	65.5	.301	*Gelidium*	0.09	0.03	0.05	0.05	0.85	7.7	73.0	.803
*Gelidium*	0.12	0.00	0.06	0.10	0.62	9.1	74.6	.829									

*Note*: Left column: Fall, Understory Full treatments. Right column: Winter, Understory Cleared treatments. Percent cover was used for the analysis of dissimilarity. The table only includes genera that cumulatively contribute >70% dissimilarity.

The *Silvetia* Canopy effect observed in the Understory Full treatments during the fall dissipated by winter (Pairwise tests: *p* = .906, Figure 6c). The canopy did not influence the understory assemblage when the understory was cleared at the start of the experiment in fall (*p* = .361, Figure 6b) but did have an effect by winter (*p* = .021, Figure 6d). Under this understory treatment, the greatest contributors to dissimilarity (listed in order of importance, Table 2) were Bare Rock (~24%), *Centroceras* (~18%), *Corallina* (~13%), *Gigartina* (~10%), and *Gelidium* (~8%). The average percent cover of Bare Rock increased in the absence of *Silvetia* (from 15.9 ± 9.9% to 43.1 ± 15.8%), while *Gigartina* and *Gelidium* decreased (from 11.1 ± 18.8% to 5.0 ± 8.2% and 8.6 ± 11.9% to 3.0 ± 5.9%, respectively). Although *Centroceras* and *Corallina* were ranked second and third in order of importance, their trends were more ambiguous. However, both slightly increased in the absence of *Silvetia* (from 16.8 ± 22.3% to 18.2 ± 17.0% and 14.8 ± 16.0% to 16.6 ± 11.4%, respectively, Table S13, Figure S4).

As a caveat to these results, prior to initiating the field manipulations in summer, a priori testing revealed that assemblages differed between Understory Full treatments (*p* = .009, Tables S8 and S9, Figure S3). Thus, the effect of *Silvetia* removal on fall, Understory Full assemblages could be confounded with the starting state of the assemblages. However, because the starting patterns of some genera were not consistently maintained across every season (e.g., *Corallina* had similar starting abundances in summer but decreased in the absence of a canopy in fall, Figure S4), it is likely that the differences found during subsequent sampling resulted from manipulating the canopy and understory rather than a holdover from the starting state of the assemblage.

## 4 DISCUSSION

Realistic assemblages of the intertidal canopy‐forming rockweed, *Silvetia*, and its understory, exhibited season‐specific responses to ocean climate change. Future climate scenarios similar to those projected by the IPCC suppressed *Silvetia* growth, reduced *Silvetia* photosynthetic efficiency (measured by quantum yield), and shifted the understory seaweed communities. These effects, however, were season‐specific; both future climate scenarios (RCP 2.6 & 4.5) indirectly influenced the understory by reducing *Silvetia* cover in summer, but only the more severe scenario (RCP 4.5) produced the same effect in the winter. Similarly, future climate reduced *Silvetia* photosynthetic efficiency in the summer but not the winter. The summertime reductions in *Silvetia* cover under future climate scenarios were then associated with shifts in the understory communities. Specifically, future climate scenarios reduced *Centroceras* and *Corallina* cover but had season‐specific impacts on *Chondracanthus* and *Laurencia* (e.g., *Chondracanthus* increased in summer but decreased in winter). Field removals of *Silvetia* similarly shifted the understory community, but only in the fall when the understory was intact.

Season‐specific impacts of climate change (e.g., in the mesocosm trials, RCP 2.6 suppressed *Silvetia* and shifted the understory during the summer trial but not in the winter trial) suggest that seasonal factors may determine how climate change affects intertidal algal communities. This phenomenon has been observed for various taxa such as insects (Johansson et al., 2020), plants (Gordo & Sanz, 2010), and migratory animals (Robinson et al., 2009) when climate change‐induced warming intersects with critical, season‐dependent phenological periods such as mating, flowering, or migration. With *Silvetia*, climate change may exacerbate mortality during the summer when it encounters temperatures near its thermal maximum, which may then reduce reproduction and recruitment in the winter (Moeller, 2002). In support of this hypothesis, *Silvetia* only grew in our mesocosms during the winter trial when photosynthetic quantum yields were higher and abiotic conditions were more benign. In summer, relative to winter, seawater pH was lower, seawater temperatures and irradiances were higher, and peak irradiance coincided more frequently with periods of low tide (an effect likely to be more pronounced in areas with larger seasonal and tidal variation; McLachlan et al., 1996), all of which may have suppressed *Silvetia* biomass during the summer trial.

The season‐specific impacts of RCP 2.6 versus the consistent impacts of RCP 4.5 on *Silvetia* suggests the potential for recovery from climate change effects if less intense climate change scenarios are realized. For example, *Silvetia* encountering biomass loss under RCP 2.6 conditions in the summer may be able to recover in the winter, though whether other processes such as reproduction will also recover remains untested. In support of this hypothesis, we only observed *Silvetia* growth in the winter trial when *Silvetia* was exposed to Ambient and RCP 2.6 climates. The realization of RCP 2.6, which hinges on extensive and immediate mitigation of greenhouse emissions, is unlikely given current trends while RCP 4.5, which calls for substantial mitigation efforts by the year 2040, appears more realistic. Consequently, the potential for recovery from climate change during at least parts of the year may be rapidly waning. However, because *Silvetia* individuals were replaced between trials, it is unclear if *Silvetia* is capable of net growth, or perhaps longer‐term acclimation when it experiences future climate conditions through consecutive seasons. More comprehensive conclusions would be drawn from experiments assessing year‐round climate change impacts on the same individuals of *Silvetia*.

Taxa resistant to direct effects of climate change may be susceptible to indirect effects via changes to canopy‐forming species (Edwards & Connell, 2012). For example, ocean acidification and warming can negatively affect canopy‐forming species (Brown et al., 2014; Shukla & Edwards, 2017) but often do not directly impact turfing algae, such as *Centroceras* (Christie et al., 2019; Ober et al., 2016). Consistent with this finding, *Centroceras* increased under future climate scenarios relative to Ambient in the absence of *Silvetia* during the winter mesocosm trial. In summer, however, *Centroceras* required a *Silvetia* canopy for survival regardless of climate treatment, potentially because ambient abiotic conditions (e.g., temperature) were too stressful during this season (Figure S1). This demonstrates how the climate‐mediated loss of canopy‐forming species may impair members of the understory assemblage which are otherwise resistant to the direct effects of climate change and that this interaction may only occur seasonally.

Understory seaweeds that are sensitive to direct impacts of ocean acidification, such as calcifying taxa like *Corallina*, may be particularly prone to climate change because of both direct (Kim et al., 2020) and indirect effects. Ocean acidification can directly reduce the growth and performance of calcifying seaweeds, in part because of reductions in calcification rates (Cornwall et al., 2022). Ocean acidification can also indirectly affect these understory species by reducing the cover provided by canopy‐forming species, thereby increasing desiccation, photoinhibition, and pH stress (Fales & Smith, 2022; Irving et al., 2004; Schmidt et al., 2011). Although we are unable to parse out all these effects here, the trend for *Corallina* loss under future climate scenarios in the presence of *Silvetia* (that occurred in both seasons) and a weak or lack of a trend in the absence of *Silvetia* suggest that some of the *Corallina* declines were indirect effects of canopy loss unrelated to an increase in photic or desiccation stress.

The effects of climate on fleshy algae such as *Laurencia* and *Chondracanthus* followed different patterns relative to turf and calcifying algae. For example, during the winter trial, *Chondracanthus* and *Laurencia* both exhibited declines under future climate scenarios relative to Ambient when without a canopy, while *Centroceras* increased under these conditions. This decline in *Laurencia* and *Chondracanthus* could have resulted from a lower thermal tolerance threshold, the lack of a biomechanism to utilize high concentrations of CO_2_ such as carbonic anhydrase, or a heavier reliance on canopies for physical and chemical amelioration (Hirsh et al., 2020; Jueterbock et al., 2013; Kim et al., 2016). These patterns, potentially driven by physiological differences and species interactions, indicate a differing response between seaweed functional groups to canopies, seasonality, and the interaction of these factors with climate change.

Under natural field conditions, assemblages also shifted in response to *Silvetia* loss depending on the season and successional stage. In the fall, the assemblages in the Understory Cleared plots did not differ between *Silvetia* Present versus Absent treatments, indicating a lack of reliance on *Silvetia* canopies by early successional species, which are generally robust to abiotic stressors (Table S12, Farrell, 1991; Sousa, 1979). The mature assemblage of Understory Full plots, however, had diverged between *Silvetia* treatments and the effect of *Silvetia* canopies on these assemblages had similarities to the mesocosm experiment (Table 2). For example, in this survey and the winter mesocosm trial, *Corallina* declined in the absence of a canopy while *Centroceras* increased. When resurveyed 2 months later in winter, the mature assemblages had homogenized, perhaps due to the recovery of species sensitive to *Silvetia* loss following cooler conditions (Cheung‐Wong et al., 2022). The assemblages within Understory Cleared plots, however, had now shifted between *Silvetia* treatments, possibly because late‐successional stage species, such as *Gelidium* and *Gigartina*, which are better competitors for space but are also reliant on canopies at higher elevations, had developed (Sousa, 1979). However, because bare rock was the primary contributor of dissimilarity, this shift may have also resulted from unrelated factors (e.g., stochastic scouring during winter storms). Regardless, if *Silvetia* cover declines under future climate conditions as seen in our mesocosm experiment, shifts in natural assemblages, such as those observed in our field experiment, will likely occur.

Climate change‐mediated shifts in the *Silvetia* assemblage will ultimately reduce or restructure intertidal communities, altering individual fitness, species interactions, and ecosystem services (Kroeker et al., 2020). Declines of *Silvetia* alone will lead to loss of nursery habitats for subtidal species during periods of submergence (Schmidt et al., 2011; Vercaemer et al., 2018) and a lack of refuge for mobile and sessile intertidal species during periods of emergence (Sapper & Murray, 2003). Loss of canopy‐forming seaweeds can also result in reduced primary production (Edwards et al., 2020; Spector & Edwards, 2020; Sullaway & Edwards, 2020), especially in the upper‐mid intertidal zone (Vadas Sr et al., 2004). Indirect effects of canopy loss will include the reduction of available habitat for understory species facilitated by canopies as well as the ecosystem services they provide (Fales & Smith, 2022). For example, future climate scenarios in our mesocosms led to decreases in *Corallina*. Because *Corallina* provides settlement cues and substrate for invertebrate larvae (Morse & Morse, 1984; Seabra et al., 2019), climate change may reduce invertebrate recruitment via changes to *Silvetia* and *Corallina*. Additionally, if the understory also facilitates a canopy‐forming species (e.g., by providing a hospitable surface for the settlement of canopy‐forming recruits), then climate‐mediated canopy loss may lead to feedback loops, causing further canopy declines and exacerbating disruption at the community level.

## 5 CONCLUSION

Our experiments considered (1) realistic assemblages that allowed for species interactions and indirect climate effects, (2) multiple future climate scenarios, and (3) seasonality. Using realistic assemblages revealed that climate change affected understory assemblages largely via indirect interactions with a canopy‐forming species. Including multiple future climate scenarios highlighted gradients in the response of *Silvetia* assemblages to increasing climate severity. Lastly, repeating our mesocosm experiment and conducting field surveys during two time periods allowed us to assess the interaction between climate change and season. Canopy‐understory interactions shape multiple communities outside of rocky intertidal habitats and it is likely for all three of the factors we tested in this experiment to be relevant for those communities. Incorporating realistic assemblages, climate scenarios, and seasonality will ultimately help better inform how important species and communities respond to climate change.

## AUTHOR CONTRIBUTIONS

**Anthony T. Truong:** Conceptualization (lead); data curation (lead); formal analysis (lead); investigation (lead); methodology (lead); project administration (lead); resources (lead); supervision (lead); writing – original draft (lead); writing – review and editing (lead). **Matthew S. Edwards:** Formal analysis (supporting); methodology (supporting); resources (supporting); validation (supporting); writing – review and editing (supporting). **Jeremy D. Long:** Conceptualization (supporting); funding acquisition (lead); investigation (supporting); methodology (supporting); project administration (supporting); resources (supporting); supervision (supporting); validation (supporting); writing – review and editing (supporting).

## CONFLICT OF INTEREST STATEMENT

The authors declare that there are no conflicts of interest.

### OPEN RESEARCH BADGES

This article has earned an Open Data badge for making publicly available the digitally‐shareable data necessary to reproduce the reported results. The data is available at http://doi.org/10.5061/dryad.05qfttf8b.

## Supporting information


Figure S1

Figure S2

Figure S3

Figure S4

Tables S1–S13

## Data Availability

The data that support the findings of this study are openly available in Dryad at http://doi.org/10.5061/dryad.05qfttf8b. Reviewer sharing link: https://datadryad.org/stash/share/5K_D9Bcf_lR‐wP4f9GkEyJpoMuCWtP9n_tsBZH32L7U.
