# Supplementary material for: Season‐specific impacts of climate change on canopy‐forming seaweed communities
Source: Ecol Evol. 2024 Feb 13;14(2):e10947. doi: 10.1002/ece3.10947 (PMC10864935; doi:10.1002/ece3.10947)
Supplement: Supplementary file 1 — Figure S1 [file ECE3-14-e10947-s001.zip › Figure S1.docx]

**Figure S1**. Daily mesocosm seawater measurements of A) summer trial temperature, B) winter trial temperature, C) summer trial pH, and D) winter trial pH across all Climate treatments.
