# Supplementary figures and images for: Season‐specific impacts of climate change on canopy‐forming seaweed communities
Source: Ecol Evol. 2024 Feb 13;14(2):e10947. doi: 10.1002/ece3.10947 (PMC10864935; doi:10.1002/ece3.10947)

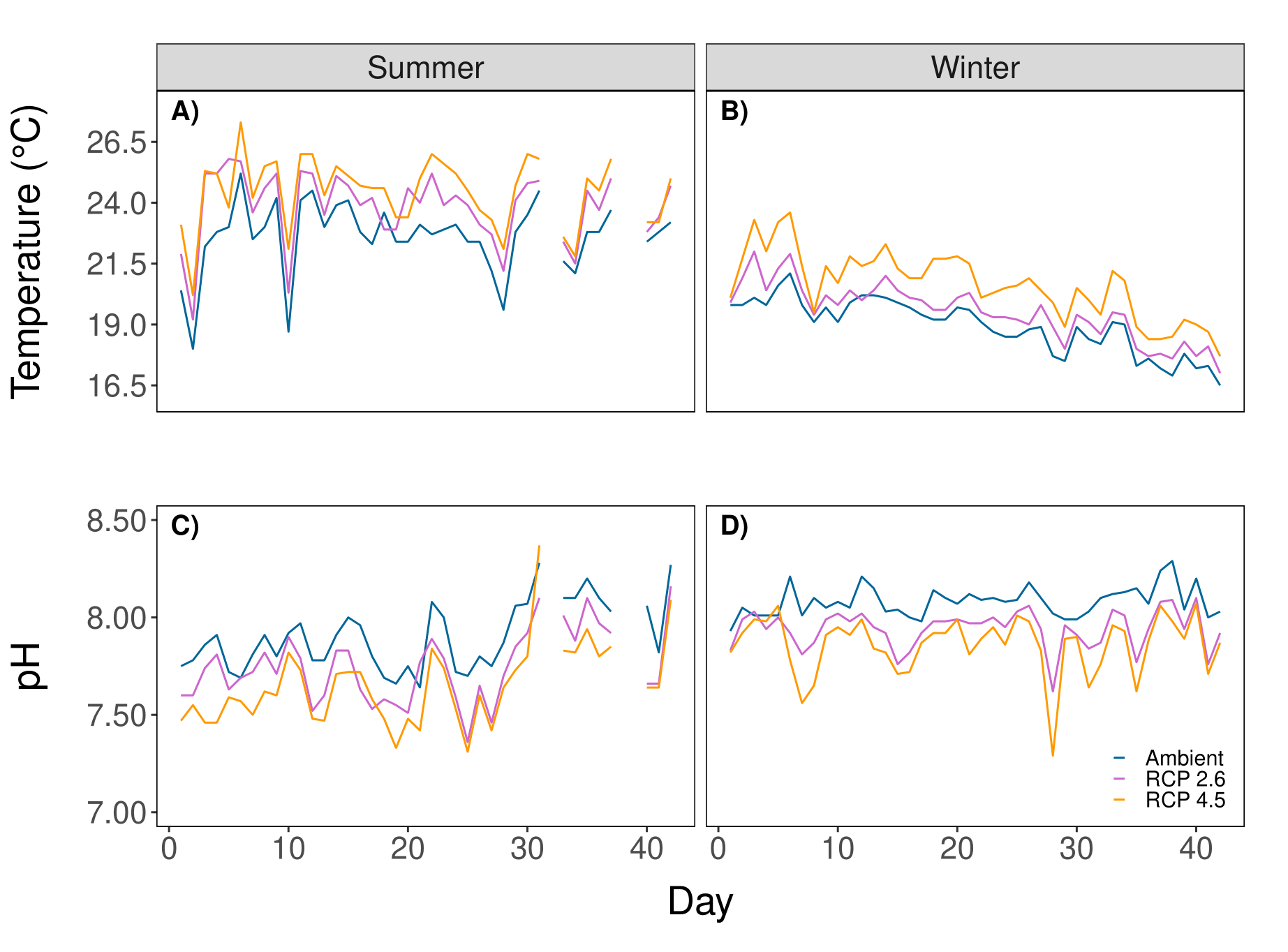

Supplement: Supplementary file 1 — Figure S1 [file ECE3-14-e10947-s001.zip › ece310947-sup-0001-FigureS1.png]

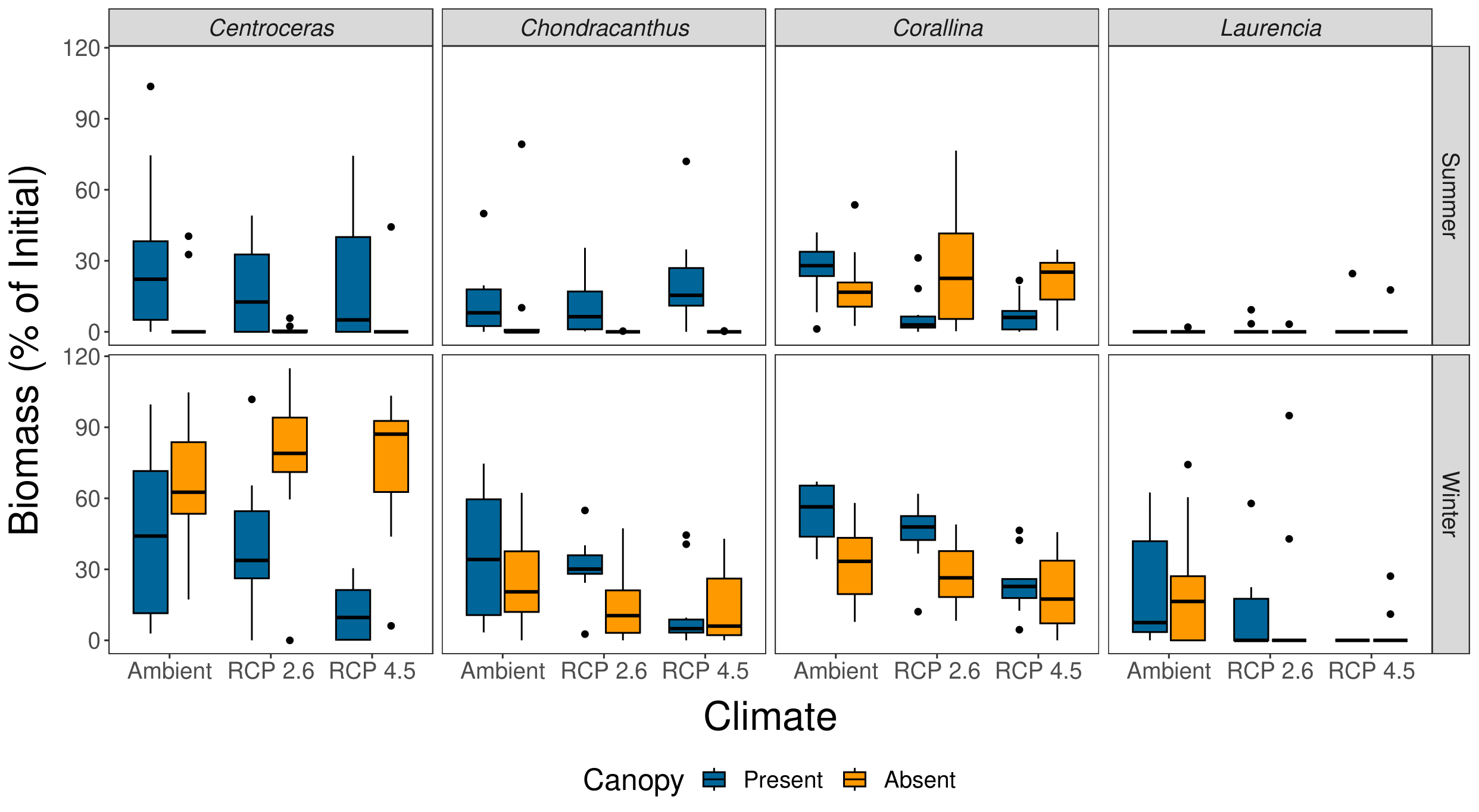

Supplement: Supplementary file 2 — Figure S2 [file ECE3-14-e10947-s003.zip › ece310947-sup-0002-FigureS2.png]

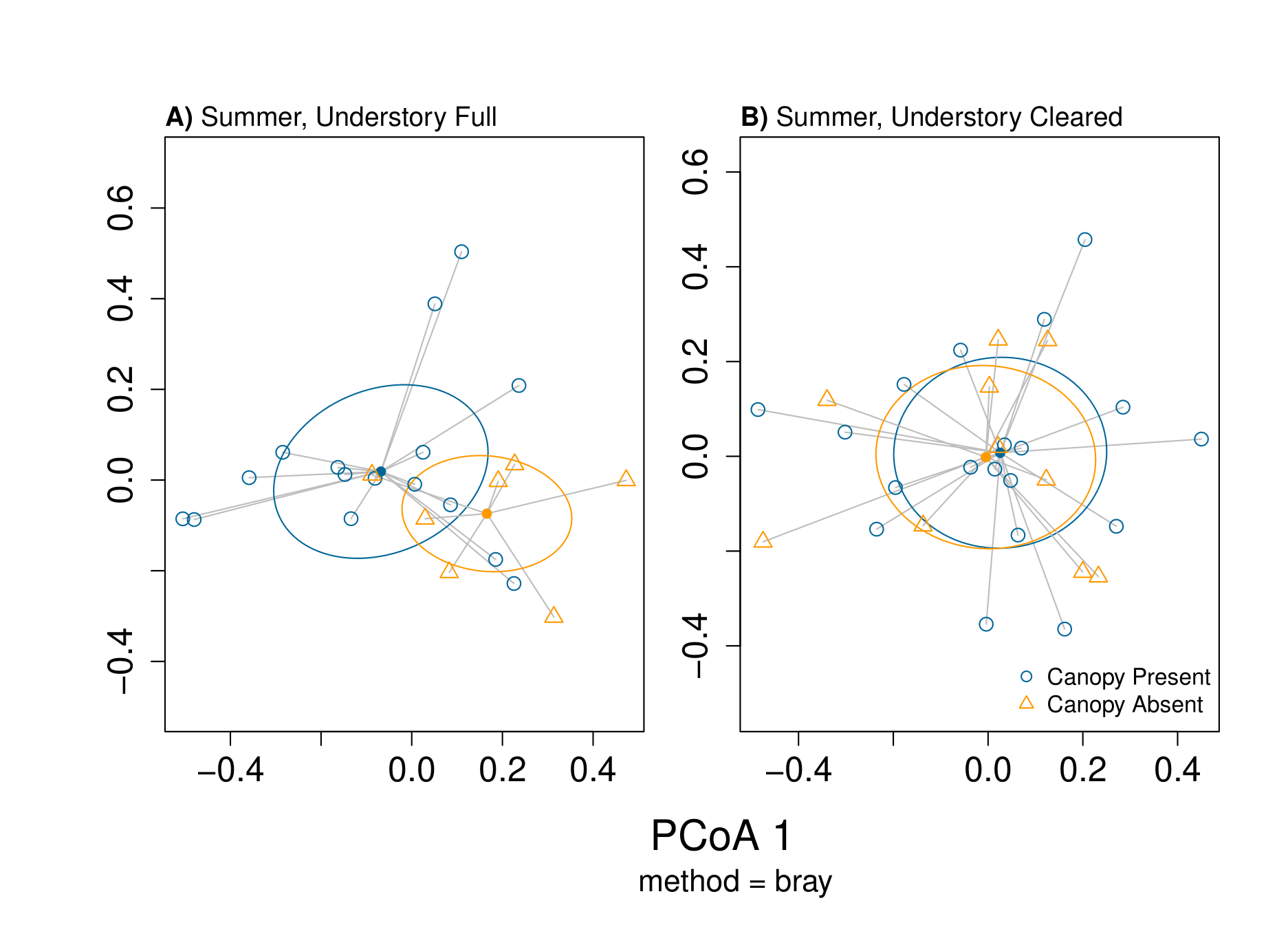

Supplement: Supplementary file 3 — Figure S3 [file ECE3-14-e10947-s005.zip › ece310947-sup-0003-FigureS3.png]

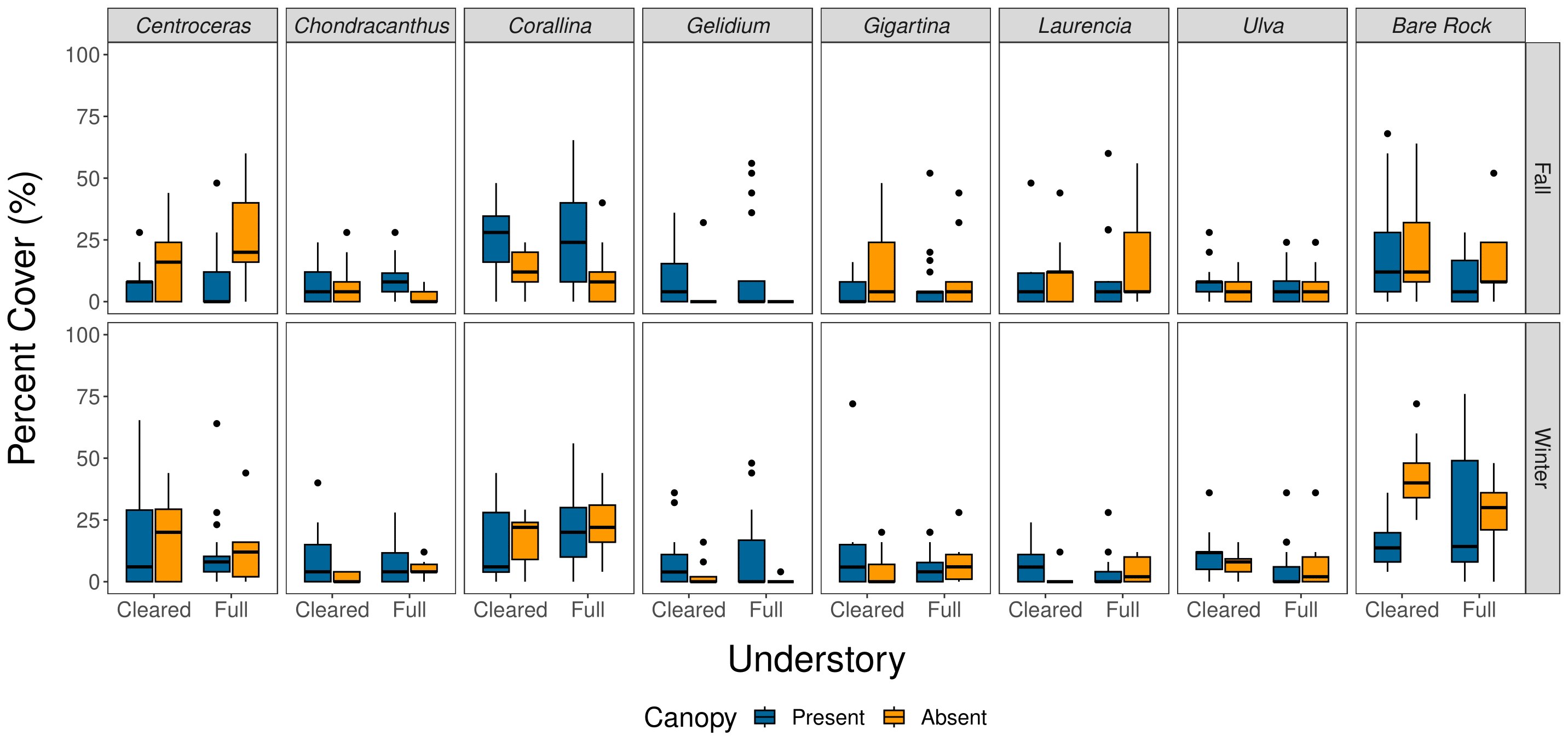

Supplement: Supplementary file 4 — Figure S4 [file ECE3-14-e10947-s004.zip › ece310947-sup-0004-FigureS4.png]
