# Supplementary material for: Season‐specific impacts of climate change on canopy‐forming seaweed communities
Source: Ecol Evol. 2024 Feb 13;14(2):e10947. doi: 10.1002/ece3.10947 (PMC10864935; doi:10.1002/ece3.10947)
Supplement: Supplementary file 2 — Figure S2 [file ECE3-14-e10947-s003.zip › Figure S2.docx]

**Figure S2**. Box and whisker plots depicting the ratio of final unbleached biomass relative to initial biomass as a percentage for each algal genera used to form simplified *Silvetia*assemblages for the Climate and *Silvetia*Canopy treatments of the mesocosm experiment. Top row from left to right: Plots depicting A) *Centroceras*, B) *Chondracanthus*, C) *Corallina*, and D) *Laurencia*relative biomass at the end of the summer trial. Bottom row from left to right: Plots depicting E) *Centroceras*, F) *Chondracanthus*, G) *Corallina*, and H) *Laurencia*relative biomass at the end of the winter trial.
