# Supplementary material for: Season‐specific impacts of climate change on canopy‐forming seaweed communities
Source: Ecol Evol. 2024 Feb 13;14(2):e10947. doi: 10.1002/ece3.10947 (PMC10864935; doi:10.1002/ece3.10947)
Supplement: Supplementary file 3 — Figure S3 [file ECE3-14-e10947-s005.zip › Figure S3.docx]

**Figure S3**. Principal Coordinates Analysis (PCoA) plots depicting natural field assemblages of designated plots prior to establishing *Silvetia*Canopy and Understory treatments in summer. A) The assemblage composition between Canopy Present and Absent treatments for Understory Full plots. B) The assemblage composition between Canopy Present and Absent treatments for Understory Cleared plots. Assemblage composition of each plot was generated using percent cover of each algal genera.
