# Supplementary material for: Season‐specific impacts of climate change on canopy‐forming seaweed communities
Source: Ecol Evol. 2024 Feb 13;14(2):e10947. doi: 10.1002/ece3.10947 (PMC10864935; doi:10.1002/ece3.10947)
Supplement: Supplementary file 4 — Figure S4 [file ECE3-14-e10947-s004.zip › Figure S4.docx]

**Figure S4**. Box and whisker plots representing average percent cover of algal genera surveyed prior to manipulations in summer and for subsequent post-manipulation surveys conducted in fall and winter, between *Silvetia*Canopy and Understory treatments. Top row from left to right: A) *Centroceras*, B) *Chondracanthus*, C) *Corallina*, D) *Gelidium*, E) *Gigartina*, F) *Laurencia*, G) *Ulva*, and H) Bare Rock percent cover in summer. Middle row from left to right: I) *Centroceras*, J) *Chondracanthus*, K) *Corallina*, L) *Gelidium*, M) *Gigartina*, N) *Laurencia*, O) *Ulva*, and P) Bare Rock percent cover in fall. Bottom row from left to right: Q) *Centroceras*, R) *Chondracanthus*, S) *Corallina*, T) *Gelidium*, U) *Gigartina*, V) *Laurencia*, W) *Ulva*, and X) Bare Rock percent cover in winter.
