# Supplementary material for: Season‐specific impacts of climate change on canopy‐forming seaweed communities
Source: Ecol Evol. 2024 Feb 13;14(2):e10947. doi: 10.1002/ece3.10947 (PMC10864935; doi:10.1002/ece3.10947)
Supplement: Supplementary file 5 — Tables S1–S13 [file ECE3-14-e10947-s002.docx]

**Table S1.** Average mesocosm temperature and pH for each Climate treatment across the 42-day duration of the A) summer and B) winter trials of the mesocosm experiment.

**A)**

| **Climate** | **Av.Temp.** | **Difference** | **Target** | **Av.pH** | **Difference** | **Target** |
| --- | --- | --- | --- | --- | --- | --- |
| Ambient | 22.6 ^°^C ± 1.48 | N/A | N/A | 7.90 ± 0.17 | N/A | N/A |
| RCP 2.6 | 23.8 ^°^C ± 1.49 | +1.2 ^°^C | +1 ^°^C | 7.74 ± 0.18 | -0.16 | -0.1 |
| RCP 4.5 | 24.4 ^°^C ± 1.44 | +1.8 ^°^C | +2 ^°^C | 7.65 ± 0.21 | -0.25 | -0.2 |

**B)**

| **Climate** | **Av.Temp.** | **Difference** | **Target** | **Av.pH** | **Difference** | **Target** |
| --- | --- | --- | --- | --- | --- | --- |
| Ambient | 18.9 ^°^C ± 1.11 | N/A | N/A | 8.08 ± 0.08 | N/A | N/A |
| RCP 2.6 | 19.5 ^°^C ± 1.16 | +0.6 ^°^C | +1 ^°^C | 7.94 ± 0.10 | -0.14 | -0.1 |
| RCP 4.5 | 20.6 ^°^C ± 1.40 | +1.8 ^°^C | +2 ^°^C | 7.86 ± 0.15 | -0.22 | -0.2 |

**Table S2.** One-way ANOVA results of Climate treatment effects on *Silvetia* biomass between the A) summer and B) winter trials of the mesocosm experiment.

**A)**

| **Source** | **Df** | **Sum Sq** | **Mean Sq** | **F-value** | **p-Value** |
| --- | --- | --- | --- | --- | --- |
| Climate | 2 | 1853.0 | 926.5 | 11.4 | < 0.001 |
| Residuals | 27 | 2198.0 | 81.4 |  |  |

**B)**

| **Source** | **Df** | **Sum Sq** | **Mean Sq** | **F-value** | **p-Value** |
| --- | --- | --- | --- | --- | --- |
| Climate | 2 | 450.8 | 225.4 | 13.4 | < 0.001 |
| Residuals | 27 | 455.1 | 16.9 |  |  |

**Table S3.** One-way ANOVA results of Climate treatment effects on *Silvetia* quantum yield between the A) summer and B) winter trials of the mesocosm experiment.

**A)**

| **Source** | **Df** | **Sum Sq** | **Mean Sq** | **F-value** | **p-Value** |
| --- | --- | --- | --- | --- | --- |
| Climate | 2 | 0.139 | 0.069 | 6.447 | 0.005 |
| Residuals | 27 | 0.290 | 0.011 |  |  |

**B)**

| **Source** | **Df** | **Sum Sq** | **Mean Sq** | **F-value** | **p-Value** |
| --- | --- | --- | --- | --- | --- |
| Climate | 2 | 0.003 | 0.001 | 0.461 | 0.635 |
| Residuals | 27 | 0.0.82 | 0.003 |  |  |

**Table S4.** Two-way PERMANOVA results of the differences in mesocosm assemblage composition between *Silvetia* Canopy and Climate treatments in the A) summer and B) winter.

**A)**

| **Source** | **Df** | **Sum Sq** | **Mean Sq** | **Pseudo-F** | **P (perm)** |
| --- | --- | --- | --- | --- | --- |
| Canopy | 1 | 896.3 | 896.3 | 15.544 | 0.001 |
| Climate | 2 | 211.2 | 105.6 | 1.831 | 0.126 |
| Ca:Cl | 2 | 317.2 | 158.6 | 2.751 | 0.029 |
| Residuals | 54 | 3113.8 | 57.7 |  |  |

**B)**

| **Source** | **Df** | **Sum Sq** | **Mean Sq** | **Pseudo-F** | **P (perm)** |
| --- | --- | --- | --- | --- | --- |
| Canopy | 1 | 1146.7 | 1146.7 | 14.343 | 0.001 |
| Climate | 2 | 988.4 | 494.2 | 6.181 | 0.001 |
| Ca:Cl | 2 | 264.2 | 132.1 | 1.652 | 0.156 |
| Residuals | 54 | 4317.3 | 80.0 |  |  |

**Table S5.** Results of *a priori* PERMANOVA post-hoc pairwise permutation tests on the similarity of mesocosm assemblage compositions between *Silvetia* Canopy and Climate treatments in A) summer and B) winter.

| **A)** |  | **Canopy Present** | | |  | **Canopy Absent** | | |
| --- | --- | --- | --- | --- | --- | --- | --- | --- |
|  |  | **t** |  | **P (perm)** |  | **t** |  | **P (perm)** |
| Ambient-RCP 2.6 |  | 2.005 |  | 0.012 |  | 1.262 |  | 0.165 |
| Ambient-RCP 4.5 |  | 2.019 |  | 0.013 |  | 1.008 |  | 0.420 |
| RCP 2.6-RCP 4.5 |  | 0.783 |  | 0.583 |  | 0.863 |  | 0.460 |

| **B)** |  | **Canopy Present** | | |  | **Canopy Absent** | | |
| --- | --- | --- | --- | --- | --- | --- | --- | --- |
|  |  | **t** |  | **P (perm)** |  | **t** |  | **P (perm)** |
| Ambient-RCP 2.6 |  | 0.804 |  | 0.001 |  | 0.775 |  | 0.831 |
| Ambient-RCP 4.5 |  | 3.483 |  | 0.001 |  | 1.539 |  | 0.065 |
| RCP 2.6-RCP 4.5 |  | 3.498 |  | 0.604 |  | 0.442 |  | 0.655 |

**Table S6.** Cumulative percent contribution, generated by SIMPER, of each algal genera to assemblage dissimilarity between Climate treatments within the *Silvetia* Canopy Absent treatment of the mesocosm experiment. Left column: Summer trial. Right column: Winter trial. The average ratio of final recovered biomass to initial biomass of each algal genera was used for analysis of dissimilarity.

**Ambient vs. RCP 2.6 (Summer, Canopy Absent) Ambient vs. RCP 2.6 (Winter, Canopy Absent)**

Average Dissimilarity: 0.16 Average Dissimilarity: 0.23

| Genus | Ambient  Av.Abund | RCP 2.6  Av.Abund | Av.Diss | SD | Ratio | Contrib.% | Cum.% | p |  | Genus | Ambient  Av.Abund | RCP 2.6  Av.Abund | Av.Diss | SD | Ratio | Contrib.% | Cum.% | p |
| --- | --- | --- | --- | --- | --- | --- | --- | --- | --- | --- | --- | --- | --- | --- | --- | --- | --- | --- |
| *Corallina* | 27.07 | 33.84 | 0.10 | 0.08 | 1.38 | 62.0 | 62.0 | 0.102 |  | *Centroceras* | 82.41 | 85.30 | 0.08 | 0.06 | 1.33 | 35.8 | 35.8 | 0.544 |
| *Chondracanthus* | 17.37 | 9.07 | 0.03 | 0.08 | 0.41 | 19.7 | 81.7 | 0.085 |  | *Laurencia* | 52.72 | 38.24 | 0.06 | 0.06 | 1.02 | 26.9 | 62.7 | 0.049 |
| *Centroceras* | 25.67 | 20.46 | 0.03 | 0.04 | 0.59 | 15.6 | 97.3 | 0.624 |  | *Chondracanthus* | 31.48 | 23.45 | 0.05 | 0.04 | 1.35 | 20.4 | 83.1 | 0.636 |
| *Laurencia* | 28.57 | 28.75 | 0.00 | 0.00 | 1.02 | 2.7 | 100.0 | 0.872 |  | *Corallina* | 39.57 | 34.68 | 0.04 | 0.03 | 1.40 | 16.9 | 100.0 | 0.970 |

**Ambient vs. RCP 4.5 (Summer, Canopy Absent) Ambient vs. RCP 4.5 (Winter, Canopy Absent)**

Average Dissimilarity: 0.15 Average Dissimilarity: 0.24

| Genus | Ambient  Av.Abund | RCP 4.5  Av.Abund | Av.Diss | SD | Ratio | Contrib.% | Cum.% | p |  | Genus | Ambient  Av.Abund | RCP 4.5  Av.Abund | Av.Diss | SD | Ratio | Contrib.% | Cum.% | p |
| --- | --- | --- | --- | --- | --- | --- | --- | --- | --- | --- | --- | --- | --- | --- | --- | --- | --- | --- |
| *Corallina* | 27.07 | 29.12 | 0.07 | 0.05 | 1.47 | 46.2 | 46.2 | 0.991 |  | *Centroceras* | 82.41 | 79.46 | 0.08 | 0.06 | 1.34 | 34.4 | 34.4 | 0.549 |
| *Centroceras* | 17.37 | 23.39 | 0.04 | 0.06 | 0.61 | 24.3 | 70.5 | 0.271 |  | *Laurencia* | 52.72 | 30.74 | 0.06 | 0.06 | 0.94 | 23.8 | 58.2 | 0.186 |
| *Chondracanthus* | 25.67 | 9.21 | 0.03 | 0.08 | 0.41 | 22.2 | 92.7 | 0.122 |  | *Corallina* | 39.57 | 28.76 | 0.05 | 0.03 | 1.49 | 20.9 | 79.1 | 0.106 |
| *Laurencia* | 28.57 | 30.12 | 0.01 | 0.02 | 0.53 | 7.3 | 100.0 | 0.517 |  | *Chondracanthus* | 31.48 | 22.16 | 0.05 | 0.04 | 1.43 | 20.9 | 100.0 | 0.362 |

**RCP 2.6 vs. RCP 4.5 (Summer, Canopy Absent) RCP 2.6 vs. RCP 4.5 (Winter, Canopy Absent)**

Average Dissimilarity: 0.13 Average Dissimilarity: 0.21

| Genus | RCP 2.6  Av.Abund | RCP 4.5  Av.Abund | Av.Diss | SD | Ratio | Contrib.% | Cum.% | p |  | Genus | RCP 2.6  Av.Abund | RCP 4.5  Av.Abund | Av.Diss | SD | Ratio | Contrib.% | Cum.% | p |
| --- | --- | --- | --- | --- | --- | --- | --- | --- | --- | --- | --- | --- | --- | --- | --- | --- | --- | --- |
| *Corallina* | 33.84 | 29.12 | 0.10 | 0.07 | 1.50 | 75.7 | 75.7 | 0.140 |  | *Centroceras* | 85.30 | 79.46 | 0.08 | 0.07 | 1.14 | 39.8 | 39.8 | 0.606 |
| *Centroceras* | 20.46 | 9.21 | 0.02 | 0.05 | 0.42 | 14.6 | 90.3 | 0.780 |  | *Corallina* | 34.68 | 28.76 | 0.05 | 0.03 | 1.43 | 22.9 | 62.7 | 0.362 |
| *Laurencia* | 28.75 | 30.12 | 0.01 | 0.02 | 0.57 | 8.9 | 99.2 | 0.086 |  | *Chondracanthus* | 23.45 | 22.16 | 0.05 | 0.04 | 1.13 | 22.6 | 85.3 | 0.691 |
| *Chondracanthus* | 9.07 | 23.39 | 0.00 | 0.00 | 1.29 | 0.8 | 100.0 | 0.995 |  | *Laurencia* | 38.24 | 30.74 | 0.03 | 0.05 | 0.62 | 14.7 | 100.0 | 0.949 |

**Table S7.** The mean and standard deviation of the ratio between recovered unbleached biomass and initial biomass for each algal genera between *Silvetia* Canopy and Climate treatments across the summer and winter trials of the mesocosm experiment.

|  |  | **Summer** | | | | | | |  | **Winter** | | | | | | |
| --- | --- | --- | --- | --- | --- | --- | --- | --- | --- | --- | --- | --- | --- | --- | --- | --- |
|  |  | **Canopy Present** | | |  | **Canopy Absent** | | |  | **Canopy Present** | | |  | **Canopy Absent** | | |
|  |  | **Ambient** | **RCP 2.6** | **RCP 4.5** |  | **Ambient** | **RCP 2.6** | **RCP 4.5** |  | **Ambient** | **RCP 2.6** | **RCP 4.5** |  | **Ambient** | **RCP 2.6** | **RCP 4.5** |
| *Centroceras* |  | 44.3 (27.5) | 33.4 (14.7) | 36.0 (21.9) |  | 25.7 (12.4) | 20.5 (1.4) | 23.4 (11.3) |  | 56.2 (27.8) | 51.6 (24.6) | 29.3 (19.6) |  | 82.4 (28.8) | 85.3 (29.0) | 79.5 (24.0) |
| *Chondracanthus* |  | 20.7 (13.6) | 19.4 (12.2) | 28.4 (18.8) |  | 17.4 (22.6) | 9.07 (0.1) | 9.2  (0.2) |  | 40.6 (25.1) | 37.4 (11.9) | 20.1 (14.8) |  | 31.5 (17.4) | 23.4 (15.3) | 22.2 (15.1) |
| *Corallina* |  | 33.4 (11.5 | 16.5 (8.9) | 16.5 (7.0) |  | 27.1 (13.5) | 33.8 (23.1) | 29.2 (9.7) |  | 58.2 (11.7) | 51.1 (12.7) | 31.5 (11.3) |  | 39.6 (14.6) | 34.7 (12.2) | 28.8 (15.4) |
| *Laurencia* |  | 28.3 (0.7) | 29.3 (2.0) | 30.1 (5.8) |  | 28.6 (0.60) | 28.7 (1.2) | 30.1 (4.1) |  | 43.4 (16.9) | 36.4 (13.4) | 28.2 (0.4) |  | 52.7 (31.7) | 38.2 (22.7) | 30.7 (6.5) |

Values are presented as mean (SD).

**Table S8.** Two-way PERMANOVA results of the differences in field assemblage composition between *Silvetia* Canopy and Understory treatments prior to manipulations in summer. Field assemblage composition was measured by percent cover surveys.

| **Source** | **Df** | **Sum Sq** | **Mean Sq** | **Pseudo-F** | **P (perm)** |
| --- | --- | --- | --- | --- | --- |
| Canopy | 1 | 2771.7 | 2771.7 | 1.715 | 0.129 |
| Understory | 1 | 1885.0 | 1885.0 | 1.166 | 0.353 |
| Ca:Un | 1 | 2056.6 | 2056.6 | 1.273 | 0.289 |
| Residuals | 50 | 80811 | 1616.2 |  |  |

**Table S9.** Results of *a priori* PERMANOVA post-hoc pairwise permutation tests on the similarity of field assemblage compositions for A) *Silvetia* Canopy and B) Understory treatments prior to manipulations in summer.

| **A)** |  | **Understory Present** | | |  | **Understory Absent** | | |
| --- | --- | --- | --- | --- | --- | --- | --- | --- |
|  |  | **t** |  | **P (perm)** |  | **t** |  | **P (perm)** |
| Canopy Present-Absent |  | 1.802 |  | 0.014 |  | 0.257 |  | 0.979 |

| **B)** |  | **Canopy Present** | | |  | **Canopy Absent** | | |
| --- | --- | --- | --- | --- | --- | --- | --- | --- |
|  |  | **t** |  | **P (perm)** |  | **t** |  | **P (perm)** |
| Understory Full-Cleared |  | 1.128 |  | 0.255 |  | 1.108 |  | 0.288 |

**Table S10.** Three-way PERMANOVA results of the differences in field assemblage composition between Season, *Silvetia* Canopy, and Understory treatments. Field assemblage composition was measured by percent cover surveys conducted in fall and winter following manipulations in summer.

| **Source** | **Df** | **Sum Sq** | **Mean Sq** | **Pseudo-F** | **P (perm)** |
| --- | --- | --- | --- | --- | --- |
| Season | 1 | 3672.20 | 3672.20 | 2.681 | 0.022 |
| Canopy | 1 | 4904.00 | 4904.00 | 3.584 | 0.002 |
| Understory | 1 | 2234.80 | 2234.80 | 1.632 | 0.160 |
| Se:Ca | 1 | 2642.70 | 2642.70 | 1.929 | 0.085 |
| Se:Un | 1 | 603.38 | 603.38 | 0.441 | 0.827 |
| Ca:Un | 1 | 405.53 | 405.53 | 0.296 | 0.910 |
| Se:Ca:Un | 1 | 1854.80 | 1854.80 | 1.354 | 0.249 |
| Residual | 84 | 115050.00 | 1369.70 |  |  |

**Table S11.** Results of PERMANOVA post-hoc pairwise permutation tests on the similarity of field assemblage compositions between A) Season, B) *Silvetia* Canopy, and C) Understory.

| **A)** |  | **Fall** | | |  | **Winter** | | |
| --- | --- | --- | --- | --- | --- | --- | --- | --- |
|  |  | **t** |  | **P (perm)** |  | **t** |  | **P (perm)** |
| Understory Full-Cleared |  | 1.361 |  | 0.110 |  | 1.161 |  | 0.250 |
| Canopy Absent-Present |  | 1.941 |  | 0.007 |  | 0.846 |  | 0.642 |

| **B)** |  | **Fall** | | | | |  | **Winter** | | | | |
| --- | --- | --- | --- | --- | --- | --- | --- | --- | --- | --- | --- | --- |
|  |  | **Understory Present** | |  | **Understory Absent** | |  | **Understory Present** | |  | **Understory Absent** | |
|  |  | **t** | **P (perm)** |  | **t** | **P (perm)** |  | **t** | **P (perm)** |  | **t** | **P (perm)** |
| Canopy Present-Absent |  | 1.886 | 0.009 |  | 1.067 | 0.361 |  | 0.499 | 0.906 |  | 1.705 | 0.021 |

| **C)** |  | **Fall** | | | | |  | **Winter** | | | | |
| --- | --- | --- | --- | --- | --- | --- | --- | --- | --- | --- | --- | --- |
|  |  | **Canopy Present** | |  | **Canopy Absent** | |  | **Canopy Present** | |  | **Canopy Absent** | |
|  |  | **t** | **P (perm)** |  | **t** | **P (perm)** |  | **t** | **P (perm)** |  | **t** | **P (perm)** |
| Understory Full-Cleared |  | 0.891 | 0.551 |  | 0.900 | 0.571 |  | 1.251 | 0.171 |  | 0.995 | 0.442 |

**Table S12.** Cumulative percent contribution for each algal genera, generated by SIMPER, to assemblage dissimilarity between *Silvetia* Canopy Present and Absent treatments in the field experiment for fall, Cleared Understory plots (left) and winter, Full Understory plots (right). Percent cover was used for analysis of dissimilarity. Table only includes genera that cumulatively contribute >70% dissimilarity.

**Canopy Present vs. Canopy Absent (Fall, Understory Cleared) Canopy Present vs. Canopy Absent (Winter, Understory Full)**

Average Dissimilarity: 0.44 Average Dissimilarity: 0.42

| Genus | Present  Av.Abund | Absent  Av.Abund | Av.Diss | SD | Ratio | Contrib.% | Cum.% | p |  | Genus | Present  Av.Abund | Absent  Av.Abund | Av.Diss | SD | Ratio | Contrib.% | Cum.% | p |
| --- | --- | --- | --- | --- | --- | --- | --- | --- | --- | --- | --- | --- | --- | --- | --- | --- | --- | --- |
| Bare Rock | 0.22 | 0.23 | 0.11 | 0.10 | 1.14 | 18.4 | 18.4 | 0.745 |  | Bare Rock | 0.26 | 0.27 | 0.12 | 0.08 | 1.55 | 21.2 | 21.2 | 0.780 |
| *Corallina* | 0.26 | 0.12 | 0.09 | 0.06 | 1.44 | 14.5 | 32.9 | 0.075 |  | *Corallina* | 0.21 | 0.21 | 0.08 | 0.06 | 1.39 | 14.8 | 36.0 | 0.729 |
| *Centroceras* | 0.07 | 0.14 | 0.07 | 0.06 | 1.23 | 11.3 | 44.2 | 0.075 |  | *Centroceras* | 0.12 | 0.14 | 0.08 | 0.08 | 1.00 | 13.6 | 49.6 | 0.373 |
| *Laurencia* | 0.08 | 0.12 | 0.06 | 0.07 | 0.95 | 10.7 | 54.9 | 0.227 |  | *Gelidium* | 0.11 | 0.01 | 0.05 | 0.08 | 0.69 | 9.5 | 59.1 | 0.939 |
| *Gigartina* | 0.04 | 0.13 | 0.06 | 0.07 | 0.92 | 10.3 | 65.2 | 0.045 |  | *Ulva* | 0.06 | 0.09 | 0.05 | 0.06 | 0.85 | 9.5 | 68.6 | 0.258 |
| *Gelidium* | 0.09 | 0.04 | 0.05 | 0.06 | 0.90 | 8.8 | 74.0 | 0.350 |  | *Gigartina* | 0.06 | 0.09 | 0.04 | 0.04 | 1.08 | 7.8 | 76.4 | 0.207 |
|  |  |  |  |  |  |  |  |  |  |  |  |  |  |  |  |  |  |  |

**Table S13.** The mean and standard deviation of the percent cover of each algal genera recorded under *Silvetia* Canopy and Understory treatments of the field experiment from surveys conducted in fall and winter. Only genera contributing to >70% dissimilarity between at least one pair of treatments were included.

|  |  | **Fall** | | | | | | |  | **Winter** | | | | | | |
| --- | --- | --- | --- | --- | --- | --- | --- | --- | --- | --- | --- | --- | --- | --- | --- | --- |
|  |  | **Understory Full** | | |  | **Understory Cleared** | | |  | **Understory Full** | | |  | **Understory Cleared** | | |
|  |  | **Canopy Present** |  | **Canopy Absent** |  | **Canopy Present** |  | **Canopy Absent** |  | **Canopy Present** |  | **Canopy Absent** |  | **Canopy Present** |  | **Canopy Absent** |
| *Centroceras* |  | 7.8 (13.7) |  | 25.3 (18.2) |  | 7.4 (8.0) |  | 14.2 (15.5) |  | 12.0 (16.0) |  | 14.0 (16.3) |  | 16.8 (22.3) |  | 18.2 (17.0) |
| *Chondracanthus* |  | 9.0 (7.9) |  | 2.2 (2.9) |  | 5.8 (7.4) |  | 7.1 (10.2) |  | 7.5 (9.2) |  | 5.3 (4.1) |  | 9.4 (11.7) |  | 1.5 (2.1) |
| *Corallina* |  | 25.5 (20.3) |  | 11.6 (13.2) |  | 26.0 (15.0) |  | 12.4 (9.3) |  | 21.0 (16.3) |  | 23.3 (14.1) |  | 14.8 (16.0) |  | 16.6 (11.4) |
| *Gelidium* |  | 12.3 (20.4) |  | 0.0 (0.0) |  | 9.2 (11.7) |  | 3.6 (10.7) |  | 10.5 (16.5) |  | 0.1 (1.6) |  | 8.6 (11.9) |  | 3.0 (5.9) |
| *Gigartina* |  | 7.1 (13.1) |  | 10.2 (16.3) |  | 4.0 (5.4) |  | 12.9 (16.3) |  | 6.0 (6.8) |  | 8.7 (10.6) |  | 11.1 (18.8) |  | 5.0 (8.2) |
| *Laurencia* |  | 10.0 (15.7) |  | 17.3 (19.8) |  | 7.7 (13.1) |  | 12.4 (14.2) |  | 3.8 (7.4) |  | 4.7 (5.9) |  | 6.8 (7.9) |  | 1.5 (4.2) |
| *Ulva* |  | 6.6 (7.0) |  | 7.1 (8.2) |  | 8.3 (8.1) |  | 5.8 (5.7) |  | 5.5 (10.0) |  | 8.7 (14.2) |  | 11.4 (9.1) |  | 7.5 (5.0) |
| Bare Rock |  | 9.2 (9.5) |  | 14.7 (16.5) |  | 21.5 (21.3) |  | 23.1 (23.0) |  | 25.8 (24.6) |  | 27.3 (16.7) |  | 15.9 (9.9) |  | 43.1 (15.8) |

Values are presented as mean (SD).
